# VDAC1-interacting proteins: binding site mapping and their derived peptides induce apoptosis and multifaceted cellular effects

**DOI:** 10.1007/s10495-025-02185-y

**Published:** 2025-09-26

**Authors:** Manikandan Santhanam, Venkatadri Babu, Anna Shteinfer-Kuzmine, Ran Zalk, Varda Shoshan-Barmaz

**Affiliations:** 1 Department of Life Sciences, Ben-Gurion University of the Negev, 84105 Beer-Sheva, Israel; 2 National Institute for Biotechnology in the Negev, Ben-Gurion University of the Negev, 84105 Beer-Sheva, Israel; 3 Ilse Katz Institute for Nanoscale Science and Technology, Ben-Gurion University of the Negev, 84105 Beer-Sheva, Israel

**Keywords:** Apoptosis, Mitochondria, Peptide array, Protein–protein interaction, VDAC1

## Abstract

The mitochondrial voltage-dependent anion channel-1 (VDAC1) protein plays a central role in regulating mitochondrial metabolism, energy production, and apoptosis. VDAC1 interacts with over 100 proteins across the cytosol, endoplasmic reticulum, plasma membrane, and mitochondrial membranes. These interactions coordinate metabolism, cell death, and signal transduction, integrating mitochondrial and cellular functions. To identify VDAC1 binding sites, we designed a peptide array of 768 peptides from 19 selected VDAC1-interacting proteins. We focused on three partners: GAPDH, gelsolin, and actin. Their VDAC1-binding sequences as peptides interacted with purified VDAC1 and, as cell-penetrating peptides, induced cell death, and elevated intracellular Ca^2^⁺ and ROS levels. Despite sequence diversity, the peptides converged on enhancing transcription factors p53 and c-Jun, upregulating VDAC1, promoting its oligomerization, and triggering apoptosis. Other effects related to their originated protein’s function include no significant effect of the GAPDH-derived peptide on its catalytic activity, indicating its effects are independent of glycolysis. The gelsolin-derived peptide altered actin organization, increasing filopodia and focal adhesion, and actin-derived peptides reduced actin, gelsolin, and tubulin expression. This study is the first to identify VDAC1 binding sites on 19 interacting partners and to demonstrate their use as cell-penetrating peptides to modulate the VDAC1 network. These findings highlight VDAC1’s multifaceted regulatory role and offer a novel approach for targeting VDAC1-protein interactions for therapeutic purposes.

**Supplementary Information:**

The online version contains supplementary material available at 10.1007/s10495-025-02185-y.

## Introduction

Mitochondria are central hubs for regulating essential cellular processes, including metabolism, the cell cycle, proliferation, differentiation, epigenetic modifications, immune signaling, apoptosis, inflammation, and aging [1]. Among mitochondrial proteins, the voltage-dependent anion channel 1 (VDAC1) plays a critical role at the crossroads of mitochondrial energy production, metabolic regulation, Ca^2^⁺ homeostasis, apoptosis, and cellular stress responses [2–4].

Due to its multifunctionality, VDAC1 has emerged as a key focus in mitochondrial research, recognized for its critical role in maintaining cellular physiology under both normal and pathological conditions. It is also increasingly viewed as a promising therapeutic target.

Located on the outer mitochondrial membrane (OMM)—the interface between the cytosol and the mitochondrial interior—VDAC1 governs the exchange of ions, nucleotides, and metabolites (such as pyruvate, malate, succinate, and NADH/NAD) up to ~ 5 kDa, and it also participates in the transport of heme and cholesterol [2, 4]. As such, VDAC1 functions as a gatekeeper, facilitating the bidirectional flow of metabolic substrates and products, thereby enabling dynamic communication between the mitochondria and the rest of the cell [2, 5, 6].

VDAC1 is now established as a key mediator of mitochondria-mediated apoptosis. It facilitates the release of pro-apoptotic proteins from the mitochondrial intermembrane space (IMS) into the cytosol, a process that critically depends on VDAC1 oligomerization [5, 7–12]. VDAC1 oligomers also enable the release of mitochondrial DNA (mtDNA) into the cytosol, linking VDAC1 to innate immune activation and inflammation [13, 14]. Given its involvement in diverse processes—including metabolism, Ca^2^⁺ regulation, apoptosis, and inflammatory responses [4, 15, 16]—VDAC1 is considered an attractive target for anticancer drug development.

Its strategic positioning at the OMM allows VDAC1 to serve as an anchoring platform for a wide range of interacting proteins from the cytosol, endoplasmic reticulum (ER), and mitochondria. Through these interactions, VDAC1 orchestrates critical metabolic and signaling pathways that support cellular energy demands or initiate programmed cell death, depending on the context [4, 15]. To date, VDAC1 has been shown to interact with more than 100 proteins, confirming its role as a central hub that links mitochondrial function to broader cellular processes [2, 4, 15, 16].

VDAC1 interacts with a wide array of proteins involved in metabolism, apoptosis, cytoskeletal organization, and signaling pathways. It interacts with metabolism-related proteins such as hexokinase (HK), c-Raf kinase, adenine nucleotide translocase (ANT) [2, 5, 10, 15], glycolytic enzyme glyceraldehyde 3-phosphate dehydrogenase (GAPDH) [17], mitochondrial creatine kinase (MtCK) [18], and carnitine palmitoyltransferase I (CPT1) [19, 20]. In the context of apoptosis, VDAC1 has been shown to interact with apoptosis-related proteins such as Bcl-2 and Bcl-xL [21], Mcl-1, Bax isoform 1 [22], Bax (apoptosis regulator BAX isoform 1) and Bim [23, 24], as well as with the translocator protein (TSPO) [25].

Moreover, VDAC1 also interacts with several cytoskeletal proteins, including gelsolin [26, 27], tubulin [28, 29], actin [29–31], and microtubule-associated protein 2 (MAP2) [32]. Additionally, it interacts with signaling proteins and chaperons such as superoxide dismutase 1 (SOD1) [33, 34], endothelial NO synthase (eNOS) [35], mitochondrial precursor, and mtHSP70/GRP75 (HSP-70) protein localized to mitochondria-associated membranes (MAMs) and forms a complex with IP3R and VDAC1 to mediate Ca^2+^ transfer from the ER to the mitochondria [36, 37]. VDAC1 also interacts with α-synuclein [38] and with the steroidogenic acute regulatory (StAR) protein, facilitating cholesterol transport into the inner mitochondrial membrane for steroidogenesis [39].

Thus, VDAC1, as a key integrator of cellular survival and death signals, is largely mediated through its protein–protein interactions (PPIs). However, most of the current knowledge about the VDAC1 interactome has been derived from indirect methods, and the specific binding sites involved in these interactions have remained largely undefined.

Recognizing the critical role of PPIs in cellular function, with alterations in these networks often linked to disease, and considering VDAC1’s multifunctionality and interactions with numerous key cellular proteins (18,19), we developed a peptide array representing 19 selected VDAC1-interacting proteins. These include mitochondrial proteins (e.g., ANT3, cytochrome c, MAVS, Bcl-2, cyclophilin D, BAD, and Mcl-1); cytoskeletal proteins (actin, tubulin α1b, and gelsolin); chaperones BiP1 and HSP-70; and other functional proteins such as GAPDH, IκBα (NF-kappa-B inhibitor alpha), and T-cell surface glycoprotein CD4 isoform 1 precursor (CD4) and CDK2 (cyclin-dependent kinase 2).

This study focuses specifically on the interaction of VDAC1 with actin, gelsolin, and GAPDH, while interactions with other proteins—including tubulin, IκBα, CD4, CDK2, BiP1, HSP-70, and various mitochondrial proteins—are reported separately.

We identified the VDAC1-binding sequences within these proteins and synthesized corresponding peptides. These peptides directly bound purified VDAC1. When produced as cell-penetrating peptides targeted to the cytosol, mitochondria, or nucleus, most, but not all, reduced cell viability and induced apoptosis.

This study is the first to systematically map VDAC1 interaction sites across 19 of its known partners and to demonstrate the functional impact of these interactions through synthetic, cell-penetrating peptides. Our findings offer a novel approach to modulate VDAC1-mediated pathways by disrupting or mimicking specific protein–protein interactions, with implications for the therapeutic targeting of mitochondrial dysfunction in disease.

## Materials and methods

### Materials

Bovine serum albumin (BSA), 4′,6-diamidino-2-phenylindole (DAPI), leupeptin, phenylmethylsulfonyl fluoride (PMSF), fluorescein isothiocyanate (FITC), propidium iodide (PI), glyceraldehyde-3-phosphate dehydrogenase (GAPDH) protein, Triton X-100, Tween-20, trypan blue, and protease inhibitor cocktail were purchased from Sigma (St. Louis, MO, USA). Paraformaldehyde was obtained from Emsdiasum (Hatfield, PA, USA). MitoSOX™ Red mitochondrial superoxide indicator and Fluo-4 AM cell permeant were obtained from Thermo Fisher (Waltham, MA, USA). A chemiluminescence EZ-ECL kit and XTT cell viability assay were from Biological Industries (Beit-Haemek, Israel). Alexa-Fluor 488-Phalloidin and a GAPDH activity assay kit (ab204732) were purchased from Abcam (Cambridge, UK). Annexin V-FITC was obtained from Alexis Biochemicals (Lausen, Switzerland). Dulbecco’s Modified Eagle’s Medium (DMEM), and Dulbecco’s phosphate-buffered saline (DPBS) normal goat serum (NGS), the supplement fetal bovine serum (FBS), and penicillin–streptomycin were obtained from Gibco (Grand Island, NY, USA). Sodium dodecyl sulfate 20% was purchased from Bio-Lab Ltd. (Jerusalem, Israel). Primary and (HRP)-conjugated and fluorophore-conjugated secondary antibodies, their sources, and the dilutions used are detailed in Table S1.

### Peptides

Customized peptides were synthesized by GL Biochem (Shanghai, China) to a level of 86–99% purity (Table S2). The peptides were dissolved in sterile double distilled water as a 10 mM solution and then diluted with sterile double distilled water to 1 mM.

### Peptide array and probing for VDAC1 interaction

A customized peptide array was produced by INTAVIS Peptide Services (GmbH & Co. KG, Tübingen, Germany). It comprised 768 peptide sequences derived from 19 VDAC1-interacting proteins that were arrayed on a glass slide. Each peptide was composed of 25 amino acids with an overlap of 15 amino acids with the peptides before and following it.

The interaction of purified VDAC1 with glass-bound peptide arrays was assessed by washing the slide three times, 10 min each, with TBST (150 mM NaCl, 50 mM Tris/HCl, pH 7.4, 0.1% Tween-20), followed by overnight incubation with blocking buffer (TBS, pH 7.8, containing low-fat dry milk, 2.5%, w/v) at 4 °C. Then slides were incubated with purified VDAC1 in protein-interacting buffer (20 mM HEPES, 100 mM KCl, pH,7.4, PBS) for 4 h at room temperature (RT). After washing, with TBST, slides were incubated overnight at 4 °C with anti-VDAC1 antibody against a sequence of 150–250 amino acids or against the VDAC1-N-terminus. Slides were then washed with TBST, followed by the appropriate secondary anti-HRP conjugated antibody for 2 h at RT. They were washed again with TBST and developed using enhanced chemiluminescence EZ-ECL (Biological Industries), according to the manufacturer’s instructions. The signal was captured in the dark using FUSION-FX (Vilber Lourmat) software.

### Microscale thermophoresis (MST)

MST analysis was performed using a NanoTemper Monolith NT.115 apparatus as described previously [4, 55]. Briefly, purified VDAC1 was fluorescently labeled using a NanoTemper Protein labeling kit BLUE (L001, NanoTemper Technologies, Munich, Germany). A constant concentration of the protein (66 nM) was incubated with different concentrations of the indicated peptide in MST binding buffer (10 mM tricine-HCl, 100 mM NaCl, pH = 7.4) for 30 min at 37 °C in the dark. Afterwards, 3–5 µl of the samples were loaded into a monolith glass capillary (NanoTemper) and a thermophoresis analysis was performed (LED 80%, IR laser 80%).

### Cell lines and culture and treatment with VDAC1-interacting CPP peptides

U-87MG (human glioblastoma; epithelial cells, ATCC: HTP-14) and PC-3 (human prostate adenocarcinoma; epithelial cells, ATCC: CRL-1435) were purchased from ATCC (Manassas, VA, USA). The cells were cultured in a humidified sterile incubator at 37 °C, with 5% CO_2_ in recommended growth medium supplemented with 10% FBS, 100 U/ml penicillin, and 100 μg/ml streptomycin. Mycoplasma contamination was routinely evaluated on cell lines. The cell lines were also authenticated by routine monitoring of cell morphology and proliferation and cultured up to 15 passages.

Cells were seeded at a density of 1 × 10^5^ cells per well in 12-well plates for apoptotic cell death analyses and at 2 × 10^5^ cells per well in 6-well plates for protein and RNA extractions. After seeding, cells were incubated for 24 h at 37 °C in a humidified atmosphere containing 5% CO₂ in serum-free medium, in the absence or presence of the indicated peptide. Following treatment, cells were collected, centrifuged at 1500 × g for 5 min, washed with phosphate-buffered saline (PBS), and processed for the designated assays.

### Cell penetration of fluorescein isothiocyanate (FITC)-labeled peptides

Cell penetration peptides (CPPs) (50 μM) were labeled with FITC by incubation for 30 min with 50 μM FITC in 10 mM Tricine buffer, pH 8.7, at 37 °C. Unreacted FITC was removed by centrifugal filtration using membranes with a cutoff of 2000 Da (Vivaspin 2, Sartorius Stedim Lab Ltd, Stonehouse, UK). U-87MG cells were incubated for 90 min with 5 μM FITC-labeled peptide in serum-free growth medium, washed with PBS, fixed with 4% paraformaldehyde, and viewed under a confocal microscope (Olympus IX81).

### Apoptotic cell death analyses

Cell death analysis was carried out using PI (0.5 µg/ml) added to the cells (6.25 μg/ml as a final concentration) with a flow cytometry iCyt sy3200 Benchtop Cell Sorter/Analyzer (Sony Biotechnology Inc., San Jose, CA, USA), using EC800 software.

Apoptotic cell death was assayed by staining with propidium iodine (PI) and FITC-annexin V, according to the manufacturer’s instructions with minor modifications. Cells were harvested and washed once with binding buffer (10 mM HEPES/NaOH, pH 7.4, 140 mM NaCl, and 2.5 mM CaCl_2_). Then cells were re-suspended in binding buffer containing FITC-annexin V and incubated for 30 min in the dark before an additional wash. PI (final concentration of 6.25 μg/ml) was added immediately before flow cytometry. At least 10,000 events were collected, recorded on a dot plot, and analyzed by flow cytometry with an iCyt sy3200 Benchtop Cell Sorter/Analyzer (Sony Biotechnology Inc.).

### Cell viability, XTT assay

Cells were analyzed for cell survival using an XTT assay, according to the manufacturer’s instructions. Briefly, the activation solution was added to the XTT solution, which is converted to orange formazan by mitochondrial dehydrogenases in live cells. Cells were then incubated for 1–2 h in the dark (5% CO_2_, 37 °C) and absorbance at 450–500 nm was analyzed using an Infinite M1000 plate reader (Tecan, Männedorf, Switzerland). Cell viability was displayed as the percentage of treated cells relative to the control ones.

### Intracellular Ca^2+^ level analysis

Fluo-4-AM (Thermo Fisher) was used to monitor changes in cytosolic Ca^2+^ levels. Cells were harvested, collected (1500×*g* for 5 min), washed with Hank’s Balanced Salt Solution (HBSS) supplemented with 1.8 mM CaCl_2_ (HBSS+), and incubated with 2 μM Fluo-4 in 200 μl HBSS(+) buffer for 30 min at 37 °C in a light-protected environment. After removing the excess dye by washing with HBSS(+), the cellular free Ca^2+^ concentration was promptly measured using an iCyt sy3200 Benchtop Cell Sorter/Analyzer. At least 10,000 events were recorded by the FL1 detector, represented as a histogram, and analyzed by EC800 software (Sony Biotechnology Inc.). Positive cells showed a shift to an enhanced level of green fluorescence (FL1).

### Reactive oxygen species (ROS) level analysis

To assess mitochondrial ROS accumulation, cells were collected and incubated with MitoSOX™ Red at 37 °C for 10 min. Fluorescence was measured using the iCyt Sy3200 Benchtop Cell Sorter/Analyzer. At least 10,000 events were recorded by the FL2 detector, represented as a histogram, and analyzed by EC800 software.

### Real-time quantitative PCR (q-RT-PCR) analysis

Total RNA was isolated from cells using the trisol reagent method. VDAC1, p53 and GAPDH mRNA quantification was carried out using specific primers (Table S3) synthesized by HyLabs (Rehovot, Israel). Real-time quantitative PCR was carried out using AB 7300 Sequence Detection Software (Applied Biosystems; Waltham, MA, USA), using SYBR Green master mix reagent Thermo Fisher Scientific (Waltham, MA, USA) according to the manufacturer’s instructions. The threshold cycle (Ct) was defined as the number of cycles required for the fluorescence intensity to rise above the background fluorescence. Relative gene expression levels were calculated using the comparative ΔΔCt method, with Ct values normalized to a reference gene and fold changes determined relative to the control condition. The mean fold changes (± SEM) of the three replicates were calculated.

### Protein extraction from cells, gel electrophoresis and immunoblotting

Cells were treated with the indicated concentrations of the peptide in serum-free growth medium for 24 h. Then cells were lysed using lysis buffer (100 mM tris/HCl (pH 8.0), 4% SDS), supplemented with a protease inhibitor cocktail (Calbiochem, San Diego, CA, USA). The lysates were then vortexed and heated at 70 °C for 10 min. Finally, cell lysates were centrifuged (15,000 g, 10 min at 4 °C), and the protein concentration of supernatant was determined according to a Lowry assay with slight modification. Protein samples were stored at -80℃ until further used for gel electrophoresis.

Protein aliquots (10–20 μg) were subjected to SDS-PAGE and then were electro-transferred onto nitrocellulose membranes for immunostaining. The membranes were incubated with a blocking solution containing 5% non-fat dry milk and 0.1% Tween-20 in TBST, followed by incubation with primary antibodies (Table S1). Subsequently, membranes were incubated with HRP-conjugated anti-mouse or anti-rabbit IgG as secondary antibodies. Band intensities were analyzed by densitometry using FUSION-FX (Vilber Lourmat) or ImageJ software, and the values were normalized to the intensities of the appropriate β-actin signal that served as a loading control.

### Chemical cross-linking

Following the designated treatment, cells (1mg/ml) were collected and incubated in PBS at pH 8.3 containing the cross-linking reagent EGS (100 μM) for 15 min at 30 °C. Samples (60 µg protein) were then subjected to SDS-PAGE, followed by immunoblotting using anti-VDAC1 antibodies. Quantitative analysis of immuno-reactive VDAC1 dimer, trimer, and multimer bands was performed using ImageJ software.

### Immunofluorescence (IF)

Cells were seeded on sterile glass coverslips in 12-well plates and cultured until reaching around 80% confluence. Cells were treated with the desired peptide in a serum-free DMEM media for 24 h. Then cells were washed with PBS, fixed with 4% paraformaldehyde (20 min), washed three times with PBS, permeabilized with 0.3% Triton X-100 in PBS (PBST), and blocked with blocking buffer (1% fatty acid-free BSA diluted in PBS) for 2 h. Cells were probed with the desired primary antibodies in an antibody solution containing 1% fatty acid-free BSA in PBST and incubated overnight at 4℃. The next day, cells were washed three times with PBS and probed with fluorescent-conjugated secondary antibody for 2 h at RT in the dark. Following a wash with PBS, coverslips were incubated with DAPI (0.5 μg/ml) for 15 min in the dark, and carefully washed, dried, and mounted on slides with Fluoroshield mounting medium (Immunobioscience, Mukilteo, WA, USA). After overnight drying at 4℃, images were acquired using a confocal microscope (Olympus 1X81, Tokyo, Japan).

### In-vitro migration and wound-healing assay

The migration of PC-3 cells treated with the gelsolin-derived peptide was assessed using a wound-healing assay and compared with that of untreated control cells. Cells were seeded in 24-well plates and grown to about 90% confluence. Thereafter, a 200-μl sterile pipette tip was used to scratch a fixed-width band in the cell monolayer. This step was followed by a 24-h incubation without serum. Wound closure was monitored using a digital camera mounted on a microscope to follow the position of the migrating front at defined times.

### GAPDH activity assay

Purified GAPDH was incubated with different concentrations of the GAPDH-derived peptides, and activity was measured using a GAPDH activity assay kit (Abcam, ab204732; Cambridge, UK) according to the provided instructions.

### Statistics and data analysis

Results are presented as the means ± SEM of results obtained from three or more independent experiments. The difference was considered statistically significant when the p-value was < 0.05 (*), < 0.01 (**), < 0.001 (***), or < 0.0001(****), as assessed by an unpaired Student’s two-tailed *t*-test.

## Results

### Identification of VDAC1 interaction sites using a peptide array composed of peptides derived from VDAC1-interacting proteins

**Fig. 1:**
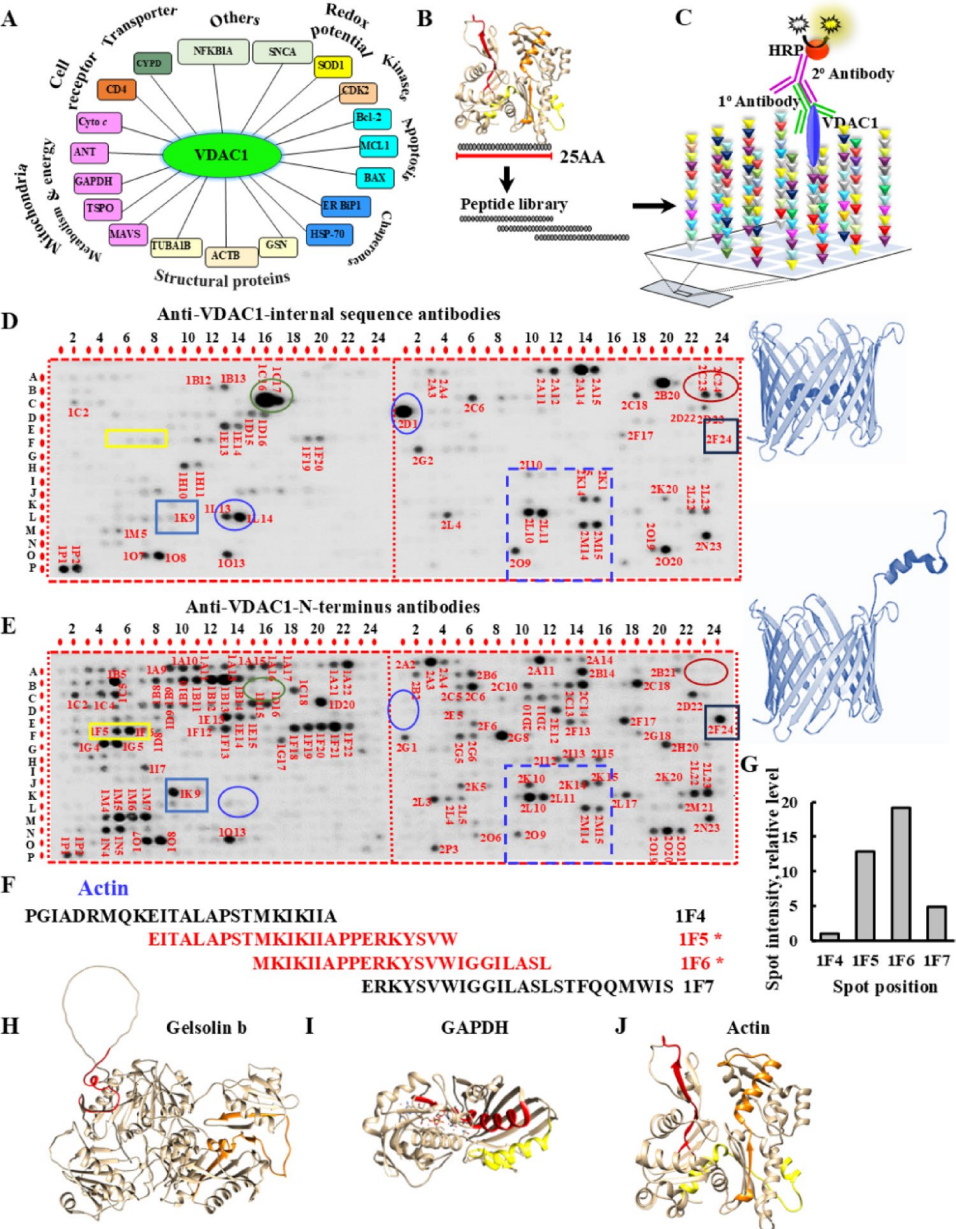
Identification of VDAC1 interaction sites in 19 VDAC1-interacting proteins using peptide arrays. **A** The selected VDAC1-interacting proteins and their function. **B**, **C** Schematic presentation of peptide arrays and detection of their interaction with VDAC1. **D**, **E** Glass-bound peptide array consisting of overlapping peptides derived from 19 VDAC1-interacting proteins were incubated 4 h with purified VDAC1 (64 nM) and then blotted with anti-VDAC1 antibodies against an internal sequence (1:5,000) (**D**) or with antibodies against the VDAC1-N-terminus (**E**), followed by incubation with HRP-conjugated anti-rabbit IgG and detection using a chemiluminescence kit. Dark spots represent the binding of VDAC1 to peptides derived from VDAC1-interacting proteins. VDAC1 3D structure with the N-terminal domain inside and outside of the channel are indicated. Also indicated are representative spots detected only by antibodies against the internal VDAC1 sequence (circled), or only by the antibodies against the N-terminus (squared), or by both antibodies (dashed line square). **F** The strongly interacting actin-derived peptides with VDAC1, spots 1F5 and 1F6, shown in red overlap by 60% with the adjacent peptides 1F4 and 1F7 (shown in black), yet no interaction with VDAC1. **G** The intensity of the peptide spots 1F4, 1F5, 1F6, and 1F7 (yellow rectangle) was analyzed using ImageJ. **H-J** 3D structures of the selected VDAC1-interacting proteins obtained from the Protein Data Bank (https://www.rcsb.org/), or when a full-length structure was not available, the AlphaFold 3D model was predicted using the database (https://alphafold.ebi.ac.uk/). Actin—AF-P12236-F1; Gelsolin—AF-P06396-F1; GAPDH—AF-P04406-F. The identified peptides were localized in each protein, and the different colors highlight different sequences (listed in Fig. S1) in the same protein interacting with VDAC1. Images were prepared with UCSF-Chimera [46] (Color figure online)

VDAC1 functions as a hub protein, engaging in interactions with numerous cellular partners involved in diverse biological processes [4, 15]. To identify the specific binding sites of VDAC1 in its partners, we selected 19 known VDAC1-interacting proteins (Fig. 1A, Table S4) and designed a glass-bound peptide array. The functions and the cellular localization of the selected VDAC1-interacting proteins, along with the published study supporting their interaction with VDAC1 are presented in Table S4. The selected proteins represent several functional groups and include: (a) *mitochondrial proteins,* some of which are *associated with apoptotic pathways*, such as TSPO, ANT3, cytochrome c (Cyto c), BAX, MAVS, cyclophilin D, BAD, and the Mcl-1 isoform 1; (b) *cytoskeletal* components, including actin, tubulin alpha 1b, and the gelsolin isoform b; (c) *chaperone* proteins such as BiP1 and HSP-70; and (d) *other proteins* such as GAPDH, IκBα, CDK-2, CD-4, α-synuclein, and SOD1.

To identify VDAC1-interaction sequences in the above proteins, we constructed a custom peptide array containing 768 peptides, each 25 amino acids in length, with a 15-amino acid overlap between consecutive peptides (Fig. 1B). To detect VDAC1 interacting peptides, the peptide array was incubated with purified VDAC1 and then with anti-VDAC1 antibodies, followed by HRP-conjugated secondary antibody that was detected by the reaction product’s chemiluminescence (Fig. 1C).

The array was probed using two different anti-VDAC1 antibodies; one produced against the N-terminal region of VDAC1 and the other recognizing an internal sequence spanning amino acids 150–250 (Fig. 1D, E; Table S5). The results revealed 74 peptides that were specifically recognized by the N-terminal antibody, 15 by the internal-sequence antibody, and 36 identified by both antibodies. The identified sequences, the protein derived from them, and their relative spot intensities indicating the strength of the interaction between VDAC1 and each peptide sequence are shown (Table S6). These findings represent the first systematic identification of VDAC1-binding sequences across multiple interacting proteins and provide a foundation for further functional studies targeting these interaction sites.

As demonstrated for selected proteins from the peptide array, distinct patterns of VDAC1 binding were observed with respect to detection by the antibodies. For example, proteins such as SOD1, GAPDH1, tubulin, and MAVS were detected only by antibodies raised against an internal VDAC1 sequence, but not with antibodies targeting the N-terminus (Table S6; Fig. 1D, E, circles). This suggests that the N-terminal domain of VDAC1 may have been engaged in binding to these specific peptides, thereby masking it from antibody recognition.

Structural studies at atomic resolution have shown that the 26-residue-long N-terminal domain of VDAC1 lies inside the pore [2]; however, it has been shown to translocate out of the pore under certain conditions [40]. These findings are in agreement with previous studies demonstrating interactions between the VDAC1 N-terminus and several proteins, including Bcl-2, Bcl-xL [9, 21, 40–43], HK-I and HK-II [9, 44], SOD1 [33], and amyloid-β (Aβ) [45].

Similarly, peptides derived from actin, MAVS, and IkB-α strongly interacted with the anti-N-terminus VDAC1 antibodies but were not detected by the antibodies against the internal VDAC1 sequence (Table S6; Fig. 1D, E, squares). This suggests that these proteins do not interact with the N-terminus, but with another region of the VDAC1 molecule.

Interestingly, certain peptides—such as those derived from BiP1 and HSP-70—were detected by both types of antibodies (Table S6; Fig. 1D, E, dashed-line square), indicating that these interaction sites are located outside both the N-terminal domain and the 150–250 internal amino-acid region, possibly in other segments of the 282-residue VDAC1 protein.

Examples of protein-derived peptides interacting with only one of the anti-VDAC1 antibodies are shown in Table S6.

The specificity of VDAC1 binding to peptide sequences is highlighted by the example of actin-derived peptides. While peptides 1F4 through 1F7 share high sequence similarity (up to 66%) (Fig. 1F), the signal intensity of 1F5, 1F6, and 1F7 are 13-, 19-, and 4.8-fold higher than that of 1F4 (Fig. 1G).

Overall, these results demonstrate that VDAC1 interacts with specific sequences derived from each of the 19 selected proteins, as detected using antibodies against an VDAC1 internal sequence and/or against the VDAC1 N-terminus (Table S5).

For certain VDAC1-interacting proteins, such as SOD, α-synuclein, a single peptide sequence was identified as directly binding to VDAC1. In contrast, for other proteins, including actin, gelsolin, and GAPDH, multiple VDAC1-binding sequences were identified that are located in different regions of the protein (Fig. S1). These binding regions are mapped within the linear amino-acid sequences of the respective proteins (Fig. S2). This pattern suggests that either VDAC1 recognizes multiple distinct interaction sites within these proteins or that the VDAC1-binding interface is formed by distant sequences that converge upon protein folding.

The identified VDAC1-binding sequences were mapped onto the three-dimensional structures of the respective proteins using data from the Protein Data Bank (PDB), AlphaFold predictions, or structural modeling in UCSF Chimera [46] where complete structural data were not available. In all cases, the VDAC1-interacting sequences (highlighted in different colors; Fig. S1) were located on the surface of the proteins, positioning them for potential interaction with VDAC1 (Fig. 1H–J).

**Fig. 2:**
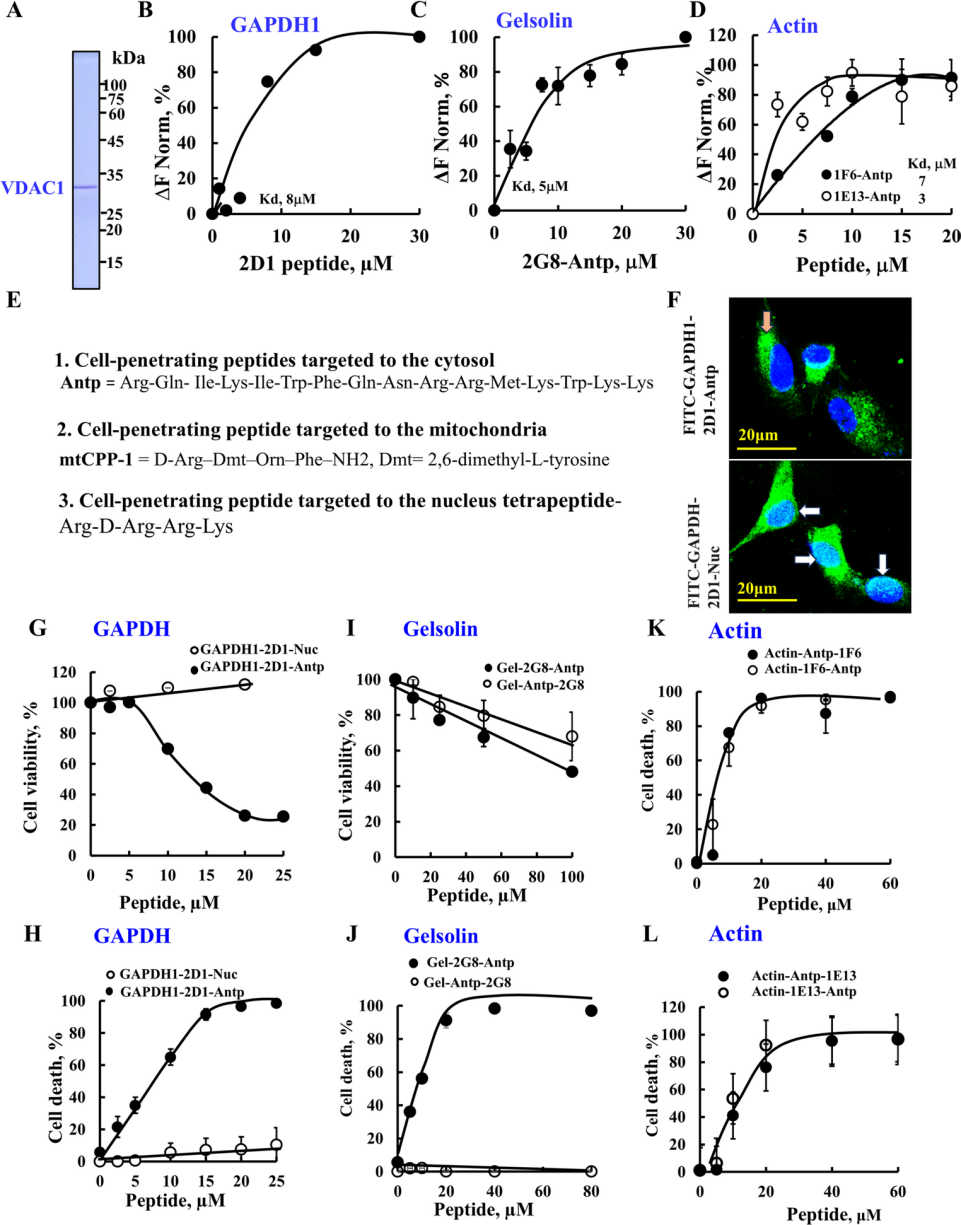
Direct binding to VDAC1 of VDAC1-interacting protein-derived peptides and when targeted to specific cellular compartments, modulate cell survival and death. Coomassie blue-stained purified VDAC1 (**A**) and its interaction with GAPDH1-2D1-derived peptide (**B**), gelsolin-2G8-Antp-derived peptide **(C)**, actin—1F6-Antp, or actin-1E13-Antp-derived peptide (**D**). Fluorescently-labeled purified VDAC1 (66 nM) was incubated for 30 min at 37 °C with the indicated synthetic VDAC1-interacting peptides (2.5–30 µM). Then MST was performed as described in the Methods section, and the obtained association constant (Kd) for each peptide is indicated. **E** The sequences added to the peptide to target them to: (**1**) the cytosol (Antp, Tf), **(2)** mitochondria (Mito), or (**3**) nucleus (Nuc) are shown. **F** U-87MG cells (100,000 cells/well, 12 well plate) seeded on coverslips were incubated for 2 h with 5 μM of FITC-labeled VDAC1-interacting peptides derived from GAPDH-2D1 and targeted to the cytosol (Antp) or the nucleus (Nuc). Cells were also stained with DAPI and visualized by confocal microscope for subcellular localization. White and orange arrows point to nuclear and cytoplasmic localization of the peptide, respectively. **G**–**L** U-87MG cells (200,000 cells/well, 6 well plate) were incubated 24 h in a serum-free medium with various concentrations of the indicated peptide: GAPDH-2D1 peptide targeted to the cytosol (Antp) or nucleus (Nuc) (0–25 μM) (**G**, **H**), gelsolin-2G8-derived peptide with Antp added to the C- or the N-terminus of the peptide (0–100 μM) (**I**, **J**), and actin-1F6 or actin-1E13-derived peptides targeted to the cytosol (Antp) or the nucleus (Nuc) (**K**, **L**) (0–60 μM)**.** Then cells were assayed for cell survival using an XTT assay (**G**, **I**) or for cell death by PI staining (**H**, **J**–**L**) (Color figure online)

To validate the peptide array findings, several VDAC1-binding sequences were synthesized as peptides and tested for direct binding to purified VDAC1 (Fig. 2A) using microscale thermophoresis (MST). Fluorescently labeled VDAC1 was incubated with increasing concentrations of each peptide, and binding was quantified. Peptides derived from GAPDH (spot 2D1, Fig. 2B), gelsolin (spot 2G8, Fig. 2C), and actin (spots 1F6 and 1E13, Fig. 2D) all demonstrated binding to VDAC1, with dissociation constants (Kd) ranging from 3 to 8 μM.

These results confirm the specificity and functional relevance of the VDAC1 interactions identified using the peptide array, highlighting the structural accessibility of these binding sites within native proteins.

### Cell penetrating peptides derived from VDAC1-interacting proteins: effects on cell viability and induction of cell death

Given the essential role of the VDAC1 interaction network in maintaining cellular homeostasis [4, 15], peptides derived from its binding partners were expected to disrupt these interactions and impact cell survival. To test this, selected VDAC1-binding sequences were produced as cell-penetrating peptides (CPPs) (Fig. 2E, Table S2).

To enable subcellular targeting, these peptides were fused to various localization sequences:

Cytosolic targeting: Peptides were fused to the Drosophila antennapedia homeodomain derived peptide (Antp), known for cell penetration, a 16-residue sequence (RQIKIWFQNRRMKWKK) [47]; Mitochondrial targeting: Peptides were linked to a mitochondria-targeting motif (mtCPP-1), composed of D-Arg–Dmt–Orn–Phe, where Dmt is 2,6-dimethyl-L-tyrosine [48]; and Nuclear targeting (nuCPP): Peptides were fused to the tetrapeptide RrRK (r = D-arginine) for nuclear localization [49].

To validate subcellular localization, GAPDH-2D1 peptides fused to Antp (cytosolic targeting) or RrRK (nuclear targeting) were labeled with FITC yielding FITC-GAPDH-2D1–Antp or FITC-GAPDH-2D1-Nuc, respectively. Confocal microscopy confirmed the targeted localization of the peptides (Fig. 2F). Because the nuclear-targeted peptide also appeared in the cytosol, its site of synthesis, its functional effects cannot be attributed exclusively to nuclear localization.

The effects of CPPs derived from GAPDH, gelsolin, and actin on cell viability, as assessed using an XTT assay, reflected cellular NADH levels, alongside apoptosis induction assays (Fig. 2G–L). The GAPDH-derived peptide, GAPDH-2D1, significantly reduced cell viability and induced apoptosis when targeted to the cytosol (IC_50,_ 12 μM) but was considerably less effective when directed to the nucleus (IC_50,_ >  > 25 μM) (Fig. 2G, H; Table S7).

The gelsolin-derived peptide (Gel-2G8), when targeted to the cytosol with Antp, regardless of whether the Antp was located at the N- or C-terminus of the peptide, weakly decreased U-87MG cell viability, as analyzed by XTT with IC_50,_ > 100 μM (Fig. 2I Table S7). However, when cell death was assayed, the peptide with the Antp added at the C-terminus induced substantial cell death (IC_50,_ 8 μM). In contrast, when Antp was localized at the N-terminus of the peptide, it induced no significant cell death (Fig. 2J, Table S7). These findings suggest that the position of the targeting sequence, whether at the N- or C-terminus of the peptide, can significantly influence the biological activity of the peptide.

VDAC1 was also found to interact with two sequences derived from actin: actin-1E13 and actin-1F6. These peptides were targeted to the cytosol using Antp at either the N- or C-terminus and assessed for their ability to induce cell death using propidium iodide (PI) staining (Fig. 2K, L, Table S7). Both peptides triggered robust cell death with IC₅₀ values of 8 μM, regardless of the position of the Antp sequence. This indicates that for these actin-derived peptides, the targeting sequence orientation does not influence their apoptotic activity.

Following these observations, we next investigated the modes of action of the most active VDAC1-binding peptides derived from GAPDH, gelsolin, and actin.

### GAPDH-2D1-derived peptide induces apoptosis via upregulation of p53 and VDAC1 expression

**Fig. 3:**
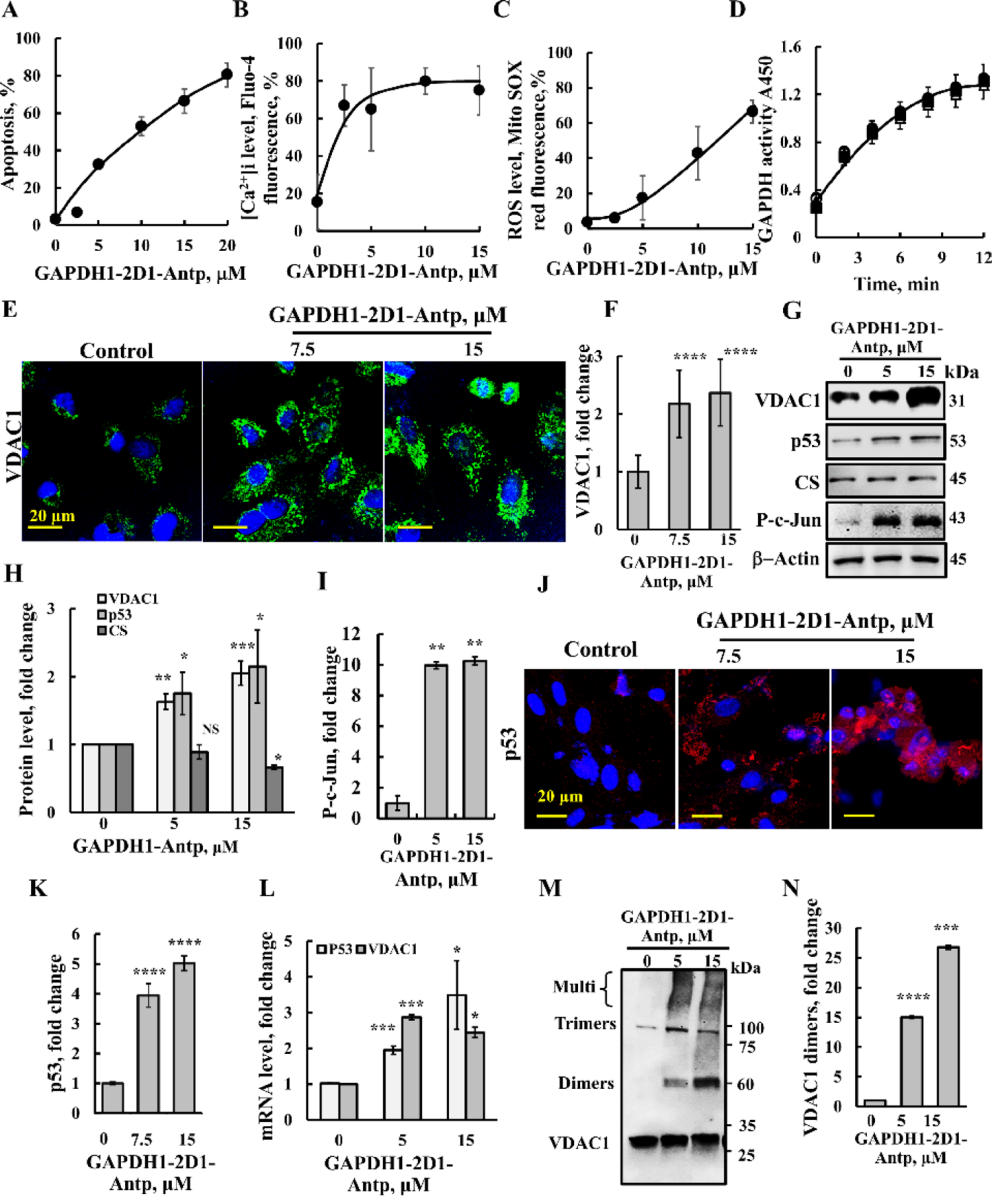
VDAC1-interacting, GAPDH-derived peptide induced apoptosis, increased intracellular Ca^2+^and ROS levels, VDAC1 expression and oligomerization, and p53 and P–c-Jun levels. **A**–**C** U-87MG cells (200,000 cells/well, 6 well plate) were incubated for 24 h with the indicated concentrations (0–20 μM) of cytosol-targeted GAPDH1-2D1-Antp peptide in a serum-free medium, followed by analysis of apoptosis using FITC–annexin V/PI staining and flow cytometry (**A**). [Ca^2+^]i levels were measured using Fluo-4 and flow cytometry (**B**), and mitochondrial superoxide levels were analyzed with MitoSOX Red and flow cytometry (**C**). **D** GAPDH enzymatic activity was assayed as described in the Methods section, following incubation for 30 min (24 °C) without (●) or with the indicated concentrations of the GAPDH1-2D1-Antp peptide (○, 5 μM; ▲, 10 μM; Δ, 20 μM; ■, 30 μM). The reaction is plotted as a function of time. **E**, **F** U-87MG cells (100,000 cells/well, 12 well plate) seeded on coverslips were incubated for 24 h in a serum-free medium with 7.5 or 15 μM of GAPDH1-2D1-Antp peptide and IF stained for VDAC1 using specific antibodies and the nucleus with DAPI (blue) (**E**), and staining intensity was quantified (**F**). **G** U-87MG cells (200,000 cells/well, 6-well plate) were incubated for 24 h in serum-free medium containing 7.5 µM or 15 µM GAPDH1-2D1-Antp peptide and then subjected to immunoblotting for VDAC1, p53, P–c-Jun, citrate synthase (CS), or β-actin, and band intensities were quantified using ImageJ software (**H**, **I**). **J**, **K** U-87MG cells were treated as in (**E**), then p53 expression levels were analyzed by IF using anti-p53 antibodies (**J**) and quantified (**K**). **L** Cells were treated as in (**G**), harvested and subjected to RNA isolation and real-time quantitative PCR of VDAC1 and p53 mRNA as described in the Materials and Methods section. **M**, **N** To monitor VDAC1 oligomerization, U-87MG cells were treated with 5 or 15 μM peptide as in **G**, harvested and subjected to cross-linking using EGS (100 μM, 1 mg protein/ml) and immunoblotting (**M**), and VDAC1 dimers were quantified (**N**). The positions of dimers, trimers, tetramers, and higher oligomers are indicated. Results represent the means ± SEM (n = 3), *p < 0.05; **p < 0.01; ***p ≤ 0.001; ****p ≤ 0.0001; NS–not significant (Color figure online)

While GAPDH is best known as a key enzyme in the glycolytic pathway, it also plays non-metabolic roles in transcriptional regulation, RNA processing, signal transduction, and apoptosis [50]. GAPDH-2D1-Antp peptide showed massive cell death (Fig. 2H) due to apoptosis induction, as demonstrated using an annexin-V/PI assay (Fig. 3A).

In addition to promoting apoptosis, the peptide also increased intracellular Ca^2+^ levels ([Ca^2+^]i), monitored using Fluo-4 and FACS analysis, and elevated ROS production, as assessed by MitoSOX Red, a mitochondrial superoxide indicator (Fig. 3B, C).

Although the peptide was derived from GAPDH, it was important to test its effect on the enzymatic activity of purified GAPDH. The results revealed no significant effect on its catalytic function (Fig. 3D), indicating that the observed biological effects are independent of GAPDH’s role in glycolysis.

Given that the GAPDH-2D1-Antp peptide was identified based on its interaction with VDAC1, we next examined whether it influenced VDAC1 expression. Surprisingly, VDAC1 protein levels were highly increased in the peptide-treated cells, as revealed by immunofluorescence staining (IF) (Fig. 3E, F), and confirmed by immunoblotting (Fig. 3G, H). In contrast, the peptide-treatment had no effect on the levels of mitochondria citrate synthase (CS) (Fig. 3G, H), suggesting that the upregulation of VDAC1 was not due to increased mitochondrial biogenesis, but rather reflected the selective elevation of VDAC1 expression within existing mitochondria.

To determine whether the peptide-induced VDAC1 overexpression was regulated by transcription factors, we examined the peptide effects on the tumor suppressor p53 and the transcription factor c-Jun, both shown to activate VDAC1 expression [51]. p53 has been reported to promote VDAC1 oligomerization [52, 53] and upregulate VDAC1 levels [54]. Immunoblotting (Fig. 3G–I) and IF staining (Fig. 3J, K) revealed that cells treated with the GAPDH-2D1-Antp peptide exhibited significantly elevated p53 expression. Similarly, phosphorylated c-Jun levels were markedly increased following peptide treatment (Fig. 3G, 3). These findings, along with the elevated levels of p53 and VDAC1 mRNA (Fig. 3L), suggest that the peptide enhances the transcription of both proteins.

Since VDAC1 overexpression, oligomerization, and apoptosis are tightly linked [6, 11, 12], we next investigated whether the GAPDH-2D1-Antp peptide influences VDAC1 oligomerization. Using chemical cross-linking with EGS and immunoblot analysis with anti-VDAC1 antibodies, we found that GAPDH-2D1-Antp cell treatment strongly induced VDAC1 oligomer formation (Fig. 3M, N).

Parallel experiments in PC-3 cells corroborated these findings: the peptide triggered cell death and enhanced both VDAC1 expression and oligomerization (Fig. S3B–E), but only when targeted to the cytosol, and not the nucleus (Fig. S3A). Consistent with the p53-null status of PC-3 cells (Fig. S3F), no p53 induction was observed; however, phosphorylated c-Jun levels were significantly elevated following peptide treatment (Fig. S3B, G, H).

Collectively, these results suggest that VDAC1’s interaction with GAPDH contributes to regulating multiple GAPDH functions beyond its role in glycolysis [17, 50].

### Cytoskeletal actin- and gelsolin-derived peptides modulate VDAC1 expression, increase filopodia, and enhance focal adhesion

**Fig. 4:**
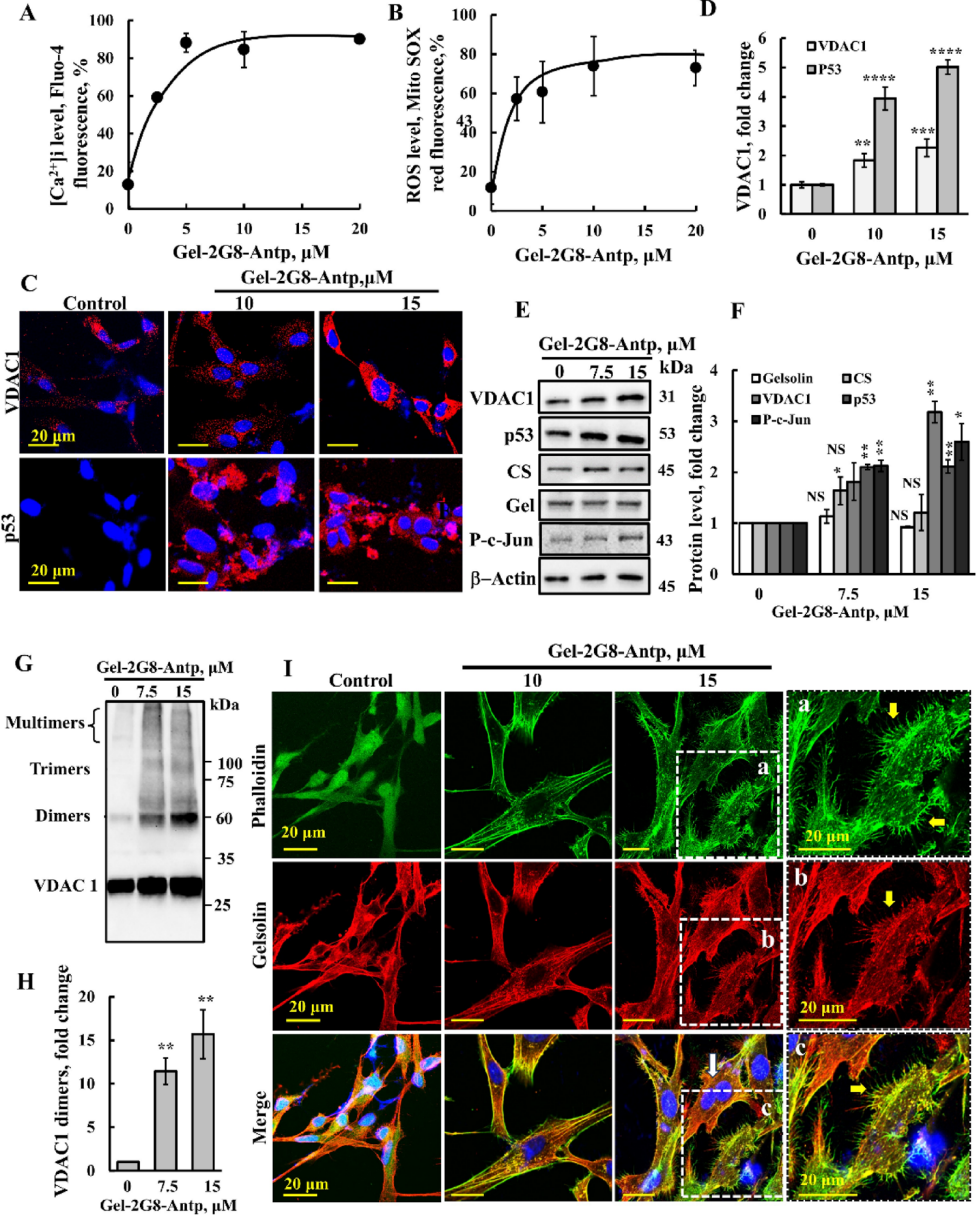
VDAC1-interacting, gelsolin-derived peptide increased intracellular Ca^2+^, ROS, VDAC1, p53, and P–c-Jun levels, induced VDAC1 oligomerization, and increased filopodia. **A**, **B** U-87MG cells (200,000 cells/well, 6 well plate) were incubated for 24 h with the indicated concentration of the Gel-2G8-Antp peptide (0–20 μM), then harvested, and [Ca^2+^]i levels were measured using Fluo-4 (**A**), and mitochondrial superoxide levels were analyzed with MitoSOX Red and flow cytometry (**B**). **C**, **D** U-87MG cells (100,000 cells/well, 12 well plate) seeded on coverslips were incubated for 24 h with 10 or 15 μM of Gel-2G8-Antp peptide and IF stained for VDAC1 or p53 using specific antibodies and the nucleus with DAPI (blue) (**C**), and staining intensity was quantified (**D**). **E**, **F** U-87MG cells (200,000 cells/well, 12-well plate) were incubated for 24 h with 7.5 or 15 μM of Gel-2G8-Antp peptide and then subjected to immunoblotting for VDAC1, p53, gelsolin, P–c-Jun, citrate synthase (CS), or β-actin (**E**), and band intensities were quantified using ImageJ software (**F**). **G**, **H** U-87MG cells were treated as in (**E**), harvested and subjected to cross-linking using EGS (100 μM, 1 mg protein/ml) to monitor VDAC1 oligomerization as revealed by immunoblotting (**G**), and VDAC1 dimers were quantified (**H**). **I** U-87MG cells were treated with the peptide as in (**C**), then co-IF stained for actin using Phalloidin-488 and for gelsolin using specific antibodies, and visualized by confocal microscopy. Selected areas were enlarged and presented at the right (**a**–**c**) to visualize actin structure filopodia. Results represent the means ± SEM (n = 3), *p < 0.05; **p < 0.01; ***p ≤ 0.001; ****p ≤ 0.0001; NS–not significant (Color figure online)

Next, we investigated the mechanism of action of a VDAC1-interacting peptide derived from gelsolin, an actin-binding protein known for severing actin filaments and regulating cell motility, shape, apoptosis, and phagocytosis [55]. Cell treatment with the Gel-2G8-Antp peptide resulted in elevated [Ca^2+^]i, and increased mitochondrial ROS production (Fig. 4A, B). Furthermore, the peptide significantly upregulated VDAC1 and p53 expressions, as demonstrated by IF (Fig. 4C, D) and an immunoblotting analysis, also showing that the peptide increased levels of activated (phosphorylated) c-Jun (Fig. 4E, F). This upregulation correlated with enhanced VDAC1 oligomerization (Fig. 4G, H) and subsequent induction of cell death (Fig. 2J).

Given gelsolin’s key role in the dynamic regulation of actin filaments through severing and capping, we examined the effect of Gel-2G8-Antp on cell morphology and actin organization (Fig. 4I). While the peptide did not significantly alter overall actin or gelsolin expression—assessed by phalloidin staining and specific antibodies, respectively (Fig. 4I)—cells treated with Gel-2G8-Antp exhibited increased filopodia formation (Fig. 4Ia–c). Notably, many peptide-treated cells displayed binucleation (Fig. 4I, white arrows; Fig. S4), suggesting that the peptide interferes with cell division.

**Fig. 5:**
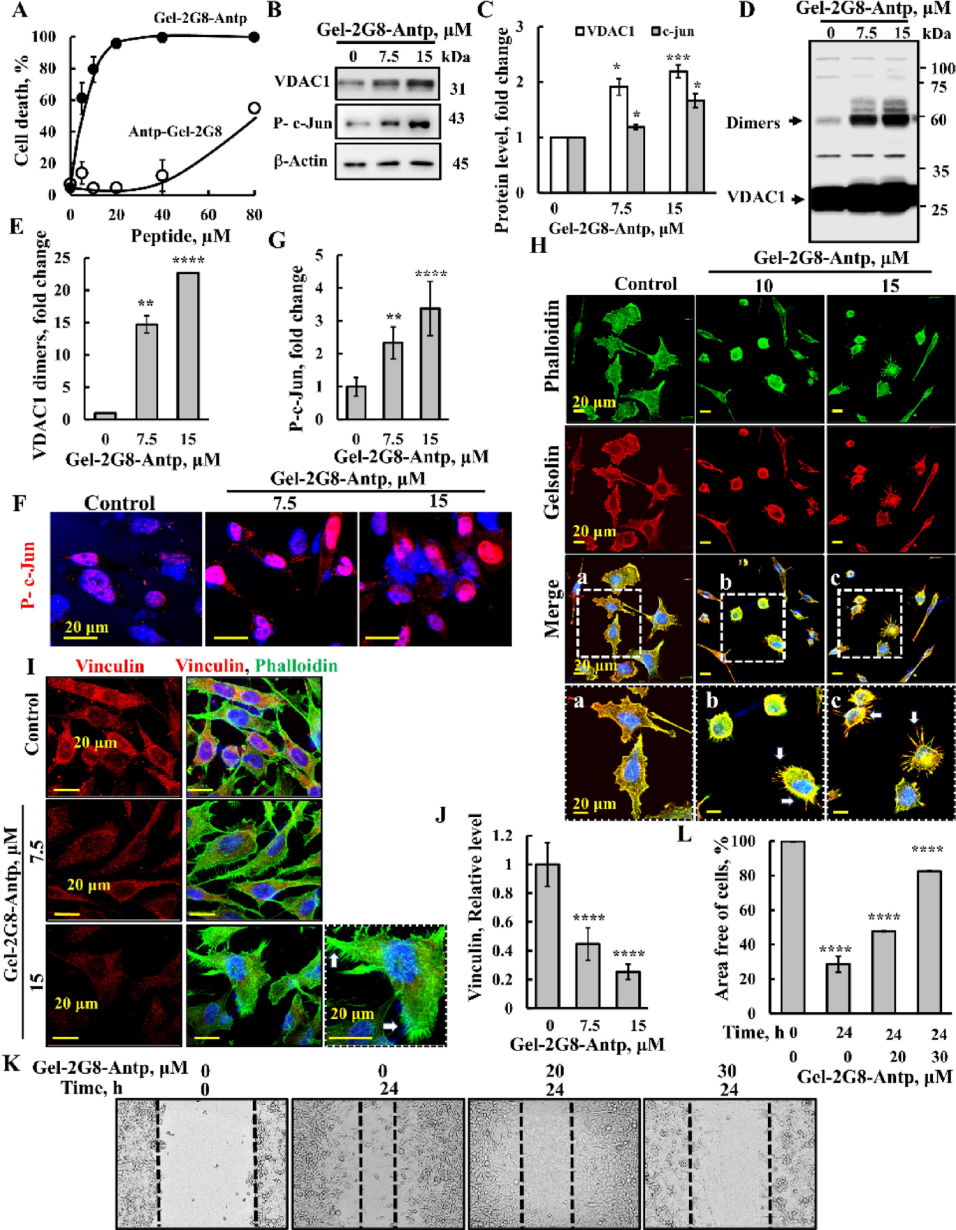
VDAC1-interacting, gelsolin-derived peptide induced cell death, filopodia formation, and focal adhesion and inhibited cell migration in PC-3 cells. **A** PC-3 cells (200,000 cells/well, 6 well plate) were incubated for 24 h in a serum-free medium with the indicated concentration (0–80 μM) of the Gel-2G8-Antp peptide or with the Antp-Gel-2G8 peptide, then harvested, and analyzed for cell death using PI staining and flow cytometry analysis. **B**, **C** PC-3 cells (200,000 cells/well, 6 well plate) were incubated with 7.5 or 15 μM of Gel-2G8-Antp peptide and then subjected to immunoblotting for VDAC1, P–c-Jun, or β-actin (**B**), and band intensities were quantified using ImageJ software (**C**). **D**, **E** PC-3 cells were treated as in (**B**), and then harvested and subjected to cross-linking using EGS (100 μM, 1 mg protein/ml) to monitor VDAC1 oligomerization as revealed by immunoblotting (**D**), and VDAC1 dimer levels were quantified (**E**). **F**, **G** PC-3 cells (100,000 cells/well, 12 well plate) seeded on coverslips were incubated for 24 h with 7.5 or 15 μM of Gel-2G8-Antp peptide, and then IF stained for P–c-Jun using specific antibodies, and the nucleus with DAPI (blue) (**F**), and staining intensity was quantified (**G**). **H** Cells were incubated with 10 or 15 μM of Gel-2G8-Antp peptide and then co-IF stained for actin using Phalloidin-488 and for gelsolin using specific antibodies, and the nucleus was stained with DAPI (blue) and visualized by confocal microscopy. Selected cells were enlarged and presented below to visualize actin filopodia. **I**, **J** Cells were treated with the peptide as in (**F**) and then IF stained for vinculin using specific antibodies and for actin with Phalloidin, with increased filopodia indicated (I, arrows), and vinculin levels were quantified (**J**). **K**, **L** Cell migration was assayed as described in the Methods section. PC-3 cells were allowed to grow to about 90% confluence. The cell layer was scraped using a 200-μl sterile pipette tip to create a scratch/wound devoid of cells. After 24 h, the scratch size was measured. Representative photomicrographs are shown (**K**) and the change in percentage of the scratch size at the indicated times is shown (**L**). Results represent the means ± SEM (n = 3), **p < 0.01; ****p ≤ 0.0001 (Color figure online)

Similar results were obtained with PC-3 cells treated with Gel-2G8-Antp peptide. Consistent with results in U-87MG cells (Fig. 2J), the peptide induced cell death when targeted to the cytosol with the Antp sequence fused to the C-terminus, but it showed a reduced effect when fused to the N-terminus (Fig. 5A). In these cells, Gel-2G8-Antp also promoted VDAC1 overexpression and oligomerization (Fig. 5B–E). Since PC-3 cells lack p53 expression (Fig. S3F), we evaluated the peptide’s impact on c-Jun phosphorylation, revealing increased levels of activated c-Jun (Fig. 5B, C, F, G).

Next, we tested the effect of the Gel-2G8-Antp peptide on the PC-3 cell morphology. Treatment with the peptide caused notable changes, as observed by altered actin and gelsolin staining patterns and co-localization, along with a marked rise in filopodia formation (Fig. 5H, arrows). Additionally, the peptide-treated cells became more rounded, likely reflecting cell death accompanied by nuclear condensation (Fig. 5H). Thus, similar to U-87MG, in PC-3 cells, the gelsolin-derived Gel-2G8-Antp peptide significantly elevated VDAC1 and c-Jun levels and promoted filopodia formation.

Actin-based structures such as lamellipodia and filopodia are critical for cell migration, largely through their connection to adhesive complexes like focal adhesions—specialized sites where clustered integrin receptors engage the extracellular matrix externally and link to actin filaments internally [56]. Therefore, we next assessed the impact of the Gel-2G8-Antp peptide on vinculin levels, a well-characterized focal adhesion protein [57]. Peptide treatment reduced vinculin expression by up to 75% compared to untreated PC-3 cells (Fig. 5I, J). Comparable reductions in vinculin levels were also observed in U-87MG cells following peptide exposure (Fig. S5). Interestingly, consistent with previous reports [58], vinculin localized as punctate structures in the PC-3 cells (Fig. 5I).

Finally, we investigated the peptide’s effect on cell migration using a wound-healing assay (Fig. 5K, L). After 24 h, untreated cells closed approximately 70% of the gap, whereas cells treated with 20 µM and 30 µM of the peptide closed only 53% and 18%, respectively, demonstrating a dose-dependent inhibition of migration.

**Fig. 6:**
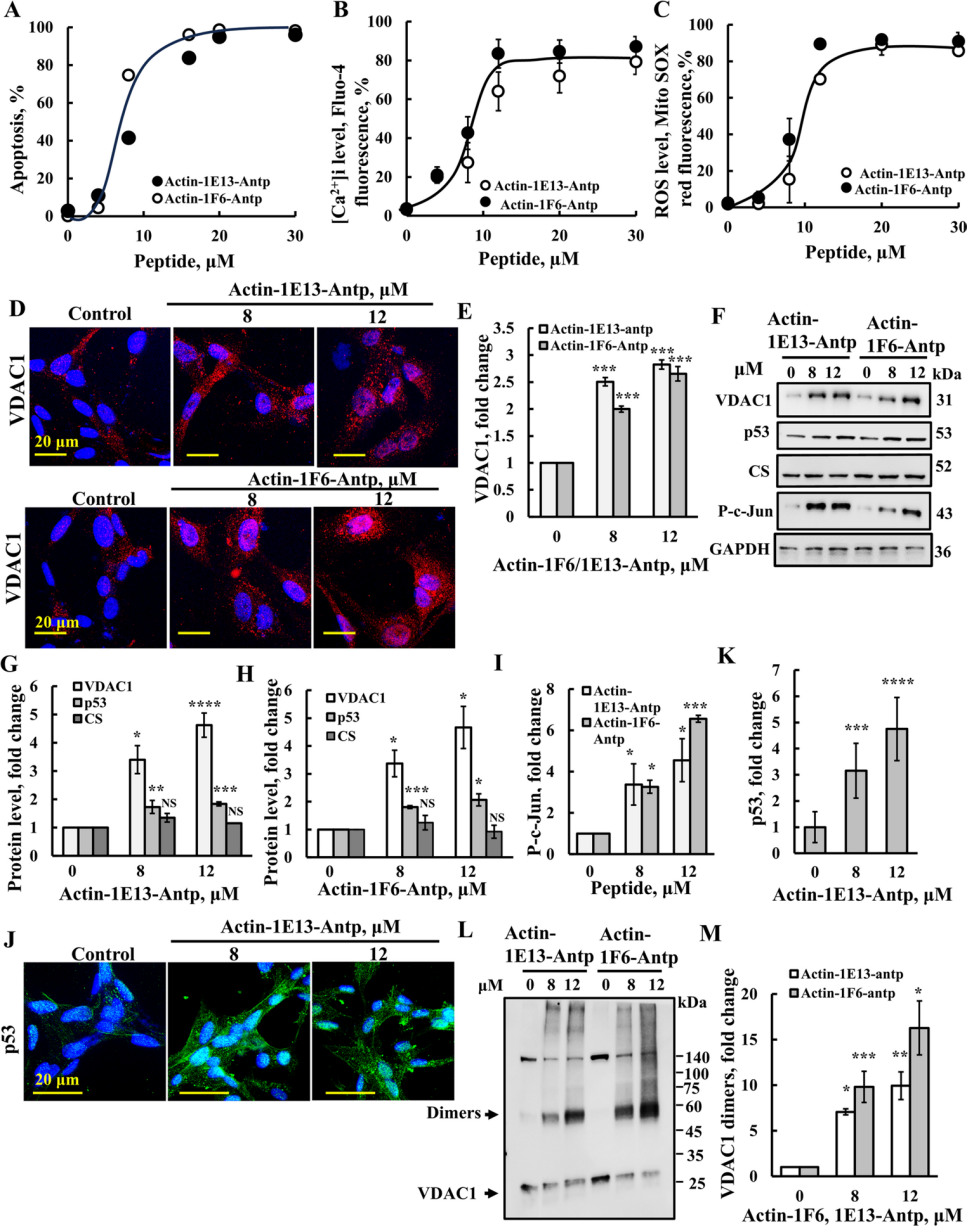
VDAC1-interacting, actin-derived peptides induced apoptosis, increased intracellular Ca^2+^, ROS, VDAC1, p53, and P–c-Jun expression levels, and triggered VDAC1 oligomerization. **A–C** U-87MG cells** (**200,000 cells/well, 6 well plate) were incubated for 24 h with the indicated concentration (0–30 μM) of the actin-derived 1E13- or 1F6-Antp, then harvested, and analyzed for apoptosis induction using FITC–annexin V/PI staining and flow cytometry analysis (**A**). [Ca.^2+^]i (**B**) and mitochondrial ROS (**C**) levels were measured using Fluo-4 and MitoSOX Red, respectively, and flow cytometry. **D**, **E** U-87MG cells (100,000 cells/well, 12 well plate) seeded on coverslips were incubated for 24 h with 8 or 12 μM of the actin-derived 1E13- or 1F6-Antp and then IF stained for VDAC1 using specific antibodies and the nucleus with DAPI (blue) (**D**), and staining intensity was quantified (**E**). **F**, **G** U-87MG cells (200,000 cells/well, 6 well plate) were incubated with 7.5 or 15 μM of the actin-derived 1E13-Antp or 1F6-Antp and then subjected to immunoblotting for VDAC1, p53, P–c-Jun, CS, or GAPDH (**F**), and band intensities were quantified using ImageJ software (**G–I**). **J**, **K** Cells were treated as in (**D**), then IF stained for p53 using specific antibodies and visualized by confocal microscopy (**J**), and intensity was quantified **(K**). **L**, **M** Cells were treated with the peptide as in (**F**), harvested and analyzed for VDAC1 oligomerization by cross-linking using EGS (100 μM, 1 mg protein/ml) and immunoblotting (**L**), and VDAC1 dimers were quantified (**M**). Results represent the means ± SEM (n = 3), **p < 0.01; ***p ≤ 0.001; ****p ≤ 0.0001 (Color figure online)

Using the peptide array, we identified three major actin sequences interacting with VDAC1: spots 1D20, 1E13/E14, and 1F5/1F6 (Figs. 1D, E, S1). CPPs derived from the 1F6 and 1E13 sequences fused with the cytosol-targeting Antp sequence at either the N- or C-terminus and induced comparable levels of cell death (Figs. 2K, L S6). Both the actin-1F6-Antp and actin-1E13-Antp peptides induced apoptosis (Fig. 6A) and increased intracellular [Ca^2+^]i and ROS levels (Fig. 6B, C).

These peptides also upregulated VDAC1, p53, and P–c-Jun expression, as demonstrated by IF and immunoblot analyses (Fig. 6D–K). Consistent with increased VDAC1 levels, the actin-derived peptides induced VDAC1 oligomerization (Fig. 6L, M).

**Fig. 7:**
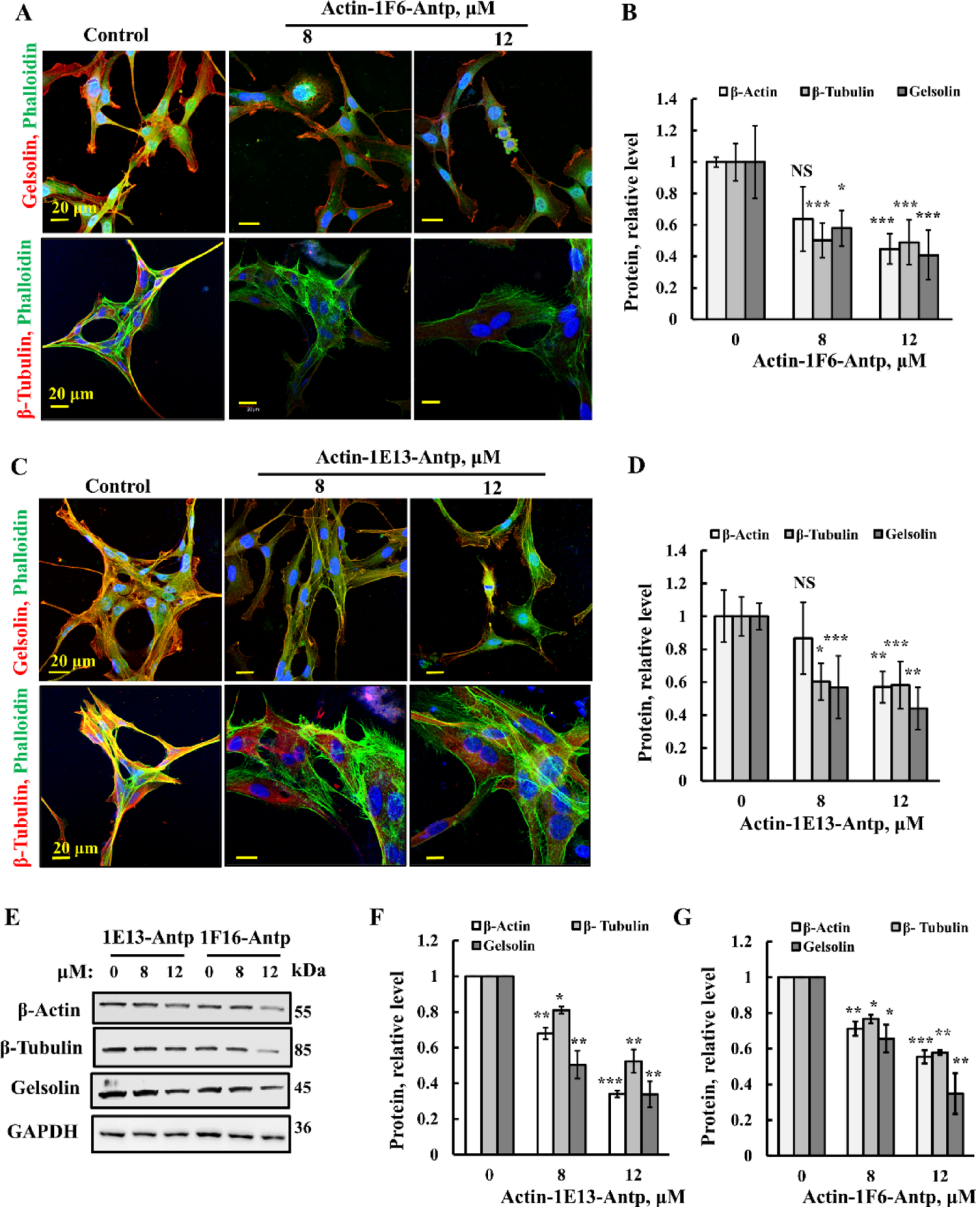
VDAC1-interacting, actin-derived peptidesreduced actin, gelsolin, and tubulin expression and induced filopodia formation (**A**–**D**) U-87MGcells were incubated for 24 h in a serum-free medium with the indicated concentration of actin-derived 1E13-Antp or 1F6-Antp and then co-IF stained for actin using Phalloidin-488 andgelsolin or β-tubulin using specific antibodies, and the nucleus with DAPI (blue), and visualized by confocal microscopy (**A** ,**C**), with staining intensity quantified (**B**, **D**). (**E**–**G**) Cells treated asabove were subjected to immunoblotting for tubulin, gelsolin, β-actin, or GAPDH (*E*), and bandintensity was quantified using ImageJ software (**F**, **G**). Results represent the means ± SEM (n = 3),**p < 0.01; ****p ≤ 0.0001 (Color figure online)

In a concentration-dependent manner, the actin-derived peptides reduced the expression of actin, gelsolin, and tubulin, as shown by phalloidin staining for actin and IF for gelsolin and tubulin (Fig. 7A–D) and confirmed by immunoblotting with specific antibodies (Fig. 7E–G).

Similar effects were observed in PC-3 cells treated with the actin-derived peptides (Fig. S7), where apoptosis, VDAC1 overexpression, and oligomerization were induced (Fig. S7A–D). Notably, these effects occurred despite the lack of p53 expression in the PC-3 cells (Fig. S3F), suggesting that VDAC1 overexpression and cell death may be mediated by increased phosphorylated c-Jun levels (Fig. S7E–H) or other transcription factors.

## Discussion

### Identification of VDAC1 interaction sites in its partner proteins and targeting of these complexes to regulate cancer cell survival

VDAC1 functions as a central hub protein, interacting with a wide range of partners to regulate diverse cellular processes [4, 15]. Despite its relatively small size (~ 32 kDa) and largely membrane-embedded structure, VDAC1 exhibits remarkable binding versatility. This is likely due to shared recognition motifs or flexible binding regions. Specifically, its N-terminus interacts with various proteins including Bcl-2 family members (e.g., Bcl-2, Bcl-xL) [9, 21, 40, 42], HK-I/HK-II [9, 44], SOD1 [33], Aβ [45], and cytoskeletal components like actin and tubulin [29].

In this study, we evaluated VDAC1’s interactions with 19 selected proteins involved in apoptosis (Bax, BAD, Mec1, Cyto c), cell cycle regulation (CDK-2), cytoskeletal functions (actin, tubulin, gelsolin), mitochondrial activity (ADP/ATP translocase, cyclophilin D, MAVS, TSPO, Cyto c), cell signaling (CD-4, IκBα, GAPDH1), and chaperone activity (BiP1, HSP-70P), as well as others including SOD1 and α-synuclein. Using a 768-peptide array and antibodies targeting the VDAC1 N-terminus or internal sequence, we mapped specific VDAC1 binding sites for each of the 19 proteins (Figs. 1D,E S1).

Interestingly, interactions with SOD1, MAVS, BiP1, tubulin-α1b, and GAPDH1 were detected only by internal-sequence antibodies (Fig. 1D, E; Tables S5, S6), suggesting that the VDAC1 N-terminus engagement in binding these partners masked it from antibody recognition. This is consistent with prior reports of N-terminal interactions with many proteins [9, 21, 29, 33, 40, 42, 43, 45],

BiP1 and HSP-70 peptides were recognized by both antibody types (Table S5, Fig. 1D, E), indicating their binding to VDAC1 outside the N-terminus and residues 150–250, likely elsewhere within the full 282-residue VDAC1.

For most proteins, a single VDAC1 binding site was identified. However, CDK-2, actin, tubulin-α1b, BiP1, HSP-70, and GAPDH1 exhibited multiple binding sequences (Figs. 1H–J, S1). Notably, all identified interaction sequences were located on the protein surface, enabling their interaction with VDAC1 (Fig. 1H–J). Additionally, synthetic peptides corresponding to these VDAC1 interaction sequences directly bind to purified VDAC1, confirming the specificity of these binding sites (Fig. 2B–D).

Peptides have recently been recognized as an attractive new modality for drug discovery, driven by advances in synthesis, modification, and analytical technologies. Our identification of VDAC1 binding sites within selected interacting proteins, and the generation of corresponding CPPs, allows the modulation of VDAC1-partner interactions, thereby influencing key cellular processes, and opening new avenues with therapeutic potential.

Most CPPs derived from VDAC1-interacting sequences affected cell viability, induced cell death, and altered signaling pathways (Figs. 2, 3, 4, 5, 6 and 7). This peptide activity depended on cellular compartment targeting the cytosol, mitochondria, or nucleus and the position of the targeting sequence, with C-terminal fusions showing greater efficacy. FITC-labeled peptides confirmed compartment-specific localization (Fig. 2F).

Interestingly, VDAC1-interacting peptides derived from GAPDH, actin, and gelsolin-induced cell death, while upregulating VDAC1, p53, and c-Jun expression, and simultaneously downregulating certain other proteins, suggesting potential modulation of the transcription factor activity, although the exact mechanisms remain unclear.

This is the first study to map VDAC1 binding sites in its partner proteins. Our results highlight VDAC1’s central role in regulating diverse cellular functions through its interactome. By disrupting VDAC1-partner interactions using the protein-derived peptides, we have demonstrated therapeutic potential.

**Fig. 8:**
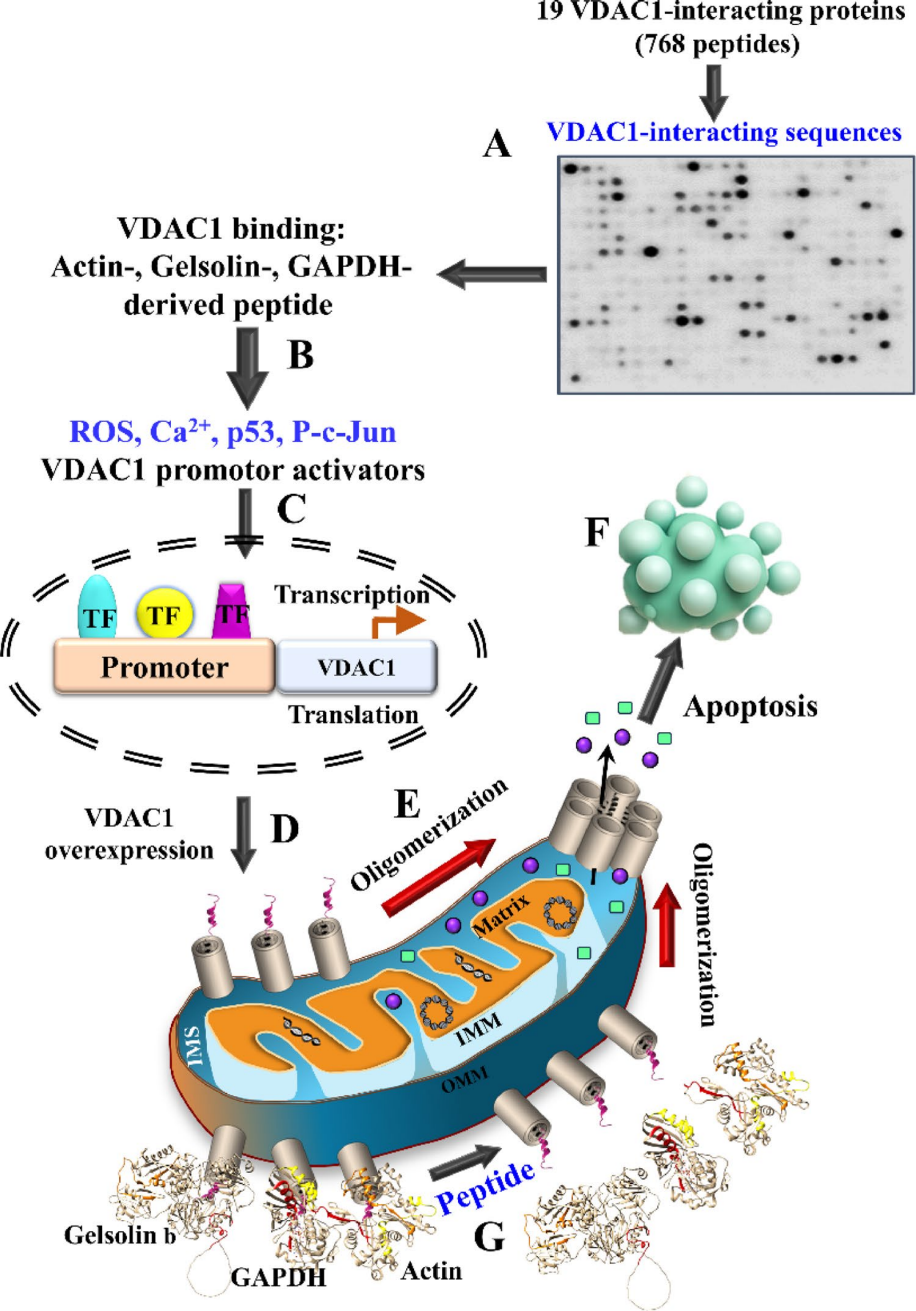
Schematic presentation of VDAC1-interacting peptides derived from GAPDH, gelsolin and actin induce inducing VDAC1 overexpression, oligomerization and apoptosis. VDAC1-interacting peptides, derived from GAPDH, gelsolin, and actin, identified via peptide array (**A**), induce mitochondria dysfunction, elevating ROS, cellular Ca^2^⁺, p53, and phosphorylated c-Jun levels (**B**). These changes activate the VDAC1 promoter (**C**), resulting in VDAC1 overexpression (**D**) and oligomerization (**E**), forming channels in the outer mitochondrial membrane that enable the release of apoptotic factors (e.g., cytochrome c, AIF) from the intermembrane space, leading to apoptosis (**F**). The peptides also displace GAPDH, gelsolin, and actin from VDAC1, facilitating its oligomerization and apoptotic function (**G**) (Color figure online)

Here, we focused primarily on three key proteins, GAPDH, gelsolin, and actin, which play critical roles in cellular functions (Fig. 8). Parallel studies on peptides derived from tubulin, CDK-2, CD4, IκBα, and eight mitochondrial proteins are currently underway and will be reported separately.

### VDAC1-interacting peptides increase p53 and activated c-Jun expression, promoting VDAC1 overexpression, oligomerization, and cell death

In this study, we showed that VDAC1-interacting peptides derived from GAPDH, actin, and gelsolin not only induced cell death, but also increased VDAC1 expression (Fig. 8). This increase in VDAC1 protein levels may result from either enhanced transcription or protein stabilization. This upregulation correlates with elevated levels of transcription factors p53 and c-Jun (Figs. 3, 4, 5 and 6). These peptides elevate the levels of these transcription factors, supporting the idea of increased VDAC1 transcription, as also reflected in the increase of mRNA levels (Fig. 3L). In p53-deficient PC-3 cells, the peptide-induced VDAC1 overexpression appears to be mediated primarily by c-Jun and possibly other transcription factors.

Thus, a key question arises: Despite their sequence diversity, how do the peptides converge on a common mechanism enhancing p53 and c-Jun expression, consequently, upregulating VDAC1? One plausible explanation is that the peptides disrupt VDAC1 function or/and its interactions, inducing cellular stress that triggers VDAC1 transcription—similar to responses observed during apoptosis induction and various pathological conditions [16]. The resulting VDAC1 overexpression drives its oligomerization (Figs. 3, 4, 5 and 6), a key step in apoptosis initiation [5, 8, 10–12].

Understanding how these VDAC1-interacting peptides modulate transcription factors levels/activity and VDAC1 expression could shed light on VDAC1 overexpression-driven pathologies and reveal novel therapeutic opportunities.

### GAPDH1-derived VDAC1-interacting peptides support GAPDH function as a multifunctional signaling protein

Beyond glycolysis, GAPDH is recognized as a multifunctional protein involving DNA repair, oxidative stress response, post-translational regulation of gene expression, and receptor-mediated signaling [17, 50, 59]. Its functions are tightly linked to its subcellular localization—regulated by post-translational modifications and its oligomeric state [50]. For example, AMPK-mediated phosphorylation of GAPDH promotes its translocation to the nucleus, where it activates Sirt1 and triggers autophagy during glucose deprivation [60]. Similarly, stress conditions drive GAPDH to the nucleus where it participates in DNA repair, RNA export, and programmed cell death [61, 62]. GAPDH also interacts with poly(ADP-ribose) polymerase (PARP) to mediate cell death [63] and activate c-Jun N-terminal kinase (JNK), triggering apoptosis via Bax translocation to the mitochondria, subsequently, inducing cell death [64].

GAPDH involvement in cellular metabolism is essential for cancer cell proliferation and tumor development across multiple cancer types [65]**,** and it also contributes to drug resistance. GAPDH is frequently upregulated in highly glycolytic tumor cells and is considered a promising target in cancer metabolism [66].

GAPDH has been implicated in diseases such as Alzheimer’s disease (AD) and other neurodegenerative disorders [67, 68] In AD, post-translationally modified GAPDH accumulates in the brain, contributing to amyloid-β amyloidogenesis [68–70]. GAPDH aggregates are released extracellularly and accumulate in amyloid plaques where both intracellular and extracellular GAPDH aggregates exert toxic effects on cells [68, 69].

Several compounds, including 3-bromopyruvate, oxamate and the natural product koningic acid, inhibit GAPDH enzymatic activity, thereby impairing glycolysis [71]. Moreover, silencing GAPDH expression with siRNA or shRNA has demonstrated antitumor effects. Several GAPDH-derived peptides were shown to enhance resistance to DNA damage in *Saccharomyces cerevisiae* cells or exhibit antimicrobial activity [71, 72] while the GDA10-3 peptide specifically shows anti-cancer properties by inhibiting cell proliferation [73]. However, their therapeutic use is limited by toxicity due to GAPDH’s essential metabolic role.

In this study, we identified a VDAC1 binding site on GAPDH (Figs. 1 and 2B). The corresponding CPP induced cell death and reduced cell viability, increased ROS and Ca^2^⁺ levels, and upregulated p53, phospho-c-Jun (P–c-Jun), and VDAC1 expression, leading to VDAC1 oligomerization and apoptosis (Figs. 2, 3 and 8). These effects align with GAPDH-mediated JNK activation [64] and our recent data linking JNK signaling to VDAC1 overexpression [51].

Importantly, unlike many GAPDH-targeting compounds, this peptide did not inhibit GAPDH enzymatic activity (Fig. 3D), suggesting that it acts as a decoy, disrupting the GAPDH–VDAC1 interaction. Since both proteins are overexpressed and vital for cancer cell survival, the VDAC1-interacting peptide derived from GAPDH offers a promising and potentially less toxic approach for cancer therapy.

### VDAC1 interactions with actin and gelsolin regulate cytoskeletal function

The cytoskeletal network comprises actin filaments, microtubules, and intermediate filaments, along with their associated regulatory proteins, all of which support key cellular functions including shape, motility, intracellular transport, and organelle dynamics [74]. Beyond these roles, actin also supports mitochondrial fission by accumulating at mitochondrial division sites [75].

Monomeric G-actin directly binds to VDAC in yeast and shown to modulate its channel activity in yeast and fungi [30, 31]. In mammals, VDAC1 directly interacts with actin [29], influencing mitochondrial dynamics, including mitochondrial movement, fusion, and fission processes [75].

Previously, we showed that a VDAC1-derived peptide (D-Δ(1–18)N-Ter-Antp) interacts with actin, altering actin/tubulin organization and inducing apoptosis, autophagy, and disrupted cell division, which results in binucleated cells [29].

Here, using the peptide array, we identified two actin-derived sequences that bind to VDAC1 and as CPPs, induce cell death (Figs. 2K, 6A, S7), These peptides increased the expression of VDAC1, p53, and c-Jun, and promoted VDAC1 oligomerization (Fig. 6D–M). Interestingly, these peptides reduced actin, gelsolin, and tubulin expression levels (Fig. 7).

Several inhibitors that target the actin cytoskeleton and influence cell structure, motility, and division have been considered for cancer and other diseases involving abnormal cell movement or division [76, 77]. However, while the actin-targeting natural compounds cytochalasin and jasplakinolide have undergone various clinical trials, particularly for cancer, their clinical application remains limited due to toxicity concerns. Additionally, latrunculins, while blocking cancer cell migration, invasion, and metastasis, face challenges related to stability, delivery, and specificity that limit their clinical use. Thymosin beta-4, which modulates inflammation and wound healing, is currently being evaluated in clinical trials for applications in tissue regeneration and inflammatory diseases. Thus, despite actin’s central role in cancer progression, no actin-targeting drugs are yet approved for chemotherapy.

Gelsolin, another key VDAC1-interacting protein, regulates actin filament severing and capping, thereby controlling actin cytoskeleton dynamics and influencing cell shape, motility, and signaling pathways (64). Its activity is regulated by intracellular calcium levels, pH, temperature, and by phosphatidylinositol-4,5-bisphosphate [55, 78].

Gelsolin interacts with VDAC1 and regulates its activity in a Ca^2+^-dependent manner [26], preventing Cyto c release and promoting cell survival [79]. Its overexpression shows anti-apoptotic effects in Alzheimer’s models [80, 81], likely through its direct binding to VDAC1 [26]. Conversely, downregulation of gelsolin expression leads to VDAC1 dimer accumulation, Cyto c release, and subsequent cell death [82]. These findings indicate that the gelsolin–VDAC1 interaction functions in apoptosis regulation, consistent with our observation that a cytosol-targeted gelsolin-derived VDAC1-interacting peptide induces extensive cell death (Figs. 2J and 5A). This effect is associated with increased phosphorylated c-Jun, p53, promoted VDAC1 overexpression, and oligomerization (Figs. 4 and 5), aligning with previous reports linking apoptosis activation to VDAC1 overexpression and oligomerization [5, 8–12] (Fig. 8).

Gelsolin-derived peptides also altered cell morphology and actin organization and promoted filopodia formation (Fig. 5H–I). Filopodia are key structures involved in environmental sensing, neurite outgrowth, cell–cell interactions, and migration [83]. This peptide inhibited cell migration (Fig. 5K, L), potentially through disrupted focal adhesions, as evidenced by reduced vinculin expression (Figs. 5I, J and S5), a well-characterized focal adhesion protein [57]. Vinculin is highly expressed in PC-3 [84] and its downregulation markedly inhibits PC-3 cell migration [85]. Its role in directional migration [86] aligns with our findings that both actin- and gelsolin-derived peptides decrease vinculin expression and suppress cell migration (Figs. 5I–L, S5). Since migration from primary tumors is a key step for cancer cell invasion and metastasis, these peptides may serve as promising agents to target metastatic cancer cells.

In cancer, gelsolin’s role is often context dependent [87–89]. Alterations in its function or expression are associated with a variety of diseases [55]. In glioblastoma, lung, breast, head and neck, gastric, and colon cancers, gelsolin has an anti-tumor function [90], and its downregulation correlates with poor patient prognosis [87–89]. In contrast, gelsolin is upregulated in prostate cancer, where it promotes progression and metastasis [91].

In neurodegenerative diseases, gelsolin mutations are associated with familial amyloidosis, a condition in which abnormal gelsolin aggregation leads to tissue damage [92]. Mutations such as D187N/Y in the Ca^2+^ binding domain 2 of gelsolin cause unfolded conformations that are susceptible to extracellular proteolytic cleavage. This generates amyloidogenic fragments (8 kDa and 5 kDa) of plasma gelsolin, which deposit systemically, leading to symptoms such as corneal lattice dystrophy and neurodegeneration [93].

Two forms of gelsolin have been identified: a 68-kDa cytosolic form, and an 86-kDa secreted (plasma) form [94]. Secreted plasma gelsolin reduced the formation of amyloid-β (Aβ40/42) peptide formation from the amyloid precursor protein (APP), indicating a neuroprotective role [95].

Modulating gelsolin interactions affects actin dynamics and key cellular processes such as motility, endocytosis, and phagocytosis [55]. Actin inhibitors can indirectly disrupt gelsolin function by altering filament integrity. Interestingly, a gelsolin-derived peptide (160-QRLFQVKGRR-169, known as PBP 10), representing the phosphoinositide-binding site, can enter cells passively. It exhibits antimicrobial activity and affects actin organization, vesicle trafficking, and other phosphoinositide-dependent eukaryotic cellular functions [96, 97].

Gelsolin and actin interactions with VDAC1 influence mitochondrial function, energy homeostasis, and apoptosis—which are critical in diseases like cancer and neurodegeneration, where VDAC plays a crucial role in regulating cell survival and death. Our results demonstrate that VDAC1-interacting peptides derived from actin and gelsolin induce cell death and disrupt the actin–gelsolin network, offering a potential strategy for cancer therapy.

In summary, VDAC1 has been found to interact with over 100 proteins involved in diverse cellular processes such as metabolism, survival, proliferation, signaling, migration, and more—functions that are essential for both normal physiology and cancer progression [4, 15]. This study is the first to identify the specific VDAC1 binding sites in 19 selected VDAC1-interacting proteins. These VDAC1 binding sites, such as CPPs, competitively disrupt VDAC1’s interactions with their target proteins, leading to inhibited cell proliferation, induced apoptosis, and alterations in other cellular functions. The wide-ranging effects of these peptides likely result from disrupting not only individual VDAC1–protein interactions, but also the broader VDAC1 protein network. These results further support VDAC1’s central regulatory role in controlling a variety of cell functions through its protein interactions. Targeting VDAC1 and its interactome by the developed peptide has promising potential for cancer treatment.

## Supplementary Information

Below is the link to the electronic supplementary material.

Supplementary Material 1

## Data Availability

No datasets were generated or analysed during the current study.
