# Supplementary material for: VDAC1-interacting proteins: binding site mapping and their derived peptides induce apoptosis and multifaceted cellular effects
Source: Apoptosis. 2025 Sep 26;30(11-12):2938–59. doi: 10.1007/s10495-025-02185-y (PMC12669278; doi:10.1007/s10495-025-02185-y)
Supplement: Supplementary file 1 — Supplementary Material 1 [file 10495_2025_2185_MOESM1_ESM.pdf]

Supplementary Data

VDAC1-interacting proteins: binding site mapping and their derived peptides induce apoptosis and multifaceted cellular effects

Manikandan Santhanam[1], Venkatadri Babu, Anna Shteinfer-Kuzmine[2], and Varda Shoshan-Barmaz[1,2]#

[1]Department of Life Sciences and the [2]National Institute for Biotechnology in the Negev, Ben-Gurion University of the Negev, Beer-Sheva 84105, Israel,

Table S1. **Antibodies used in this study**
Antibodies against the indicated protein, their catalogue number, source, and the dilutions used in immunoblotting (WB) and immunofluorescence (IF) experiments are presented below.

Antibody	Source and catalog number	Dilution	
		WB	IF
Goat anti-Mouse-HRP	Abcam, Cambridge, UK, ab97040	1:10,000	-
Goat anti-Rabbit-HRP	Promega, Madison, WI, W4011	1:15,000	-
Goat anti-Rabbit H&L-Alexa Fluor 555	Abcam, Cambridge, UK, ab150078	-	1:750
Goat anti-Rabbit H&L-Alexa Fluor 488	Abcam, Cambridge, UK, ab150077	-	1:750
Goat anti-Mouse H&L-Alexa Fluor 555	Abcam, Cambridge, UK, ab150113	-	1:750
Goat anti-Mouse H&L-Alexa Fluor 488	Abcam, Cambridge, UK, ab150114	-	1:750
Rabbit polyclonal anti-citrate synthetase	Abcam, Cambridge, UK, ab96600	1:2000	-
Rabbit Monoclonal anti-VDAC1	Abcam, Cambridge, UK, ab154856	1:10000	1:500
Mouse Monoclonal anti-VDAC1	Abcam, Cambridge, UK, ab186321		1:500
Rabbit polyclonal anti-P-c-Jun (Ser63)	Abcam, Cambridge, UK, ab31419	1:2000	-
Rabbit-monoclonal anti-vinculin	Abcam, Cambridge, UK, ab129002	-	1:1000
Rabbit polyclonal anti-actin	Abcam, Cambridge, UK, ab8227	1:40000	-
Mouse monoclonal anti-gelsolin	BD Transduction Laboratories, 610412	-	1:200
Mouse monoclonal anti-p53	Santa Cruz, sc-126	1:1000	1:1000
Mouse monoclonal anti-p53	Abcam, Cambridge, UK, ab131442	-	1:1000

Table S2. VDAC1-interacting proteins, their cell targeted derived peptides and their purity
Peptides and cell-penetrating peptides sequence, the protein derived from, the spot number, molecular mass and purity are indicated. The targeting sequence directing the peptide to the cytosol (Antp), mitochondria (Mito), or nucleus (Nuc) are indicated. The bold letters indicated the targeting sequence.

Protein name	Peptide name	Cell-penetrating peptide	MW, kDa	Purity %
**Glyceraldehyde-3-phosphate dehydrogenase isoform 1 (GAPDH1)**	2D1	NEFGYSNRVVDLMAHMASKE	2298.55	99.07
	2D1-Nuc	NEFGYSNRVVDLMAHMASKE-**R-[D Arg]-RK**	2895.54	89.76
	2D1-Antp	NEFGYSNRVVDLMAHMASKE-**RQIKIWFQNRRMKWKK**	4527.27	89.57
	2G8-Antp	MVVEHPEFLKAGKEPGLQIWRVEKF-**RQIKIWFQNRRMKWKK**	5197.30	95.88
	Antp-2G8	**RQIKIWFQNRRMKWKK**-MVVEHPEFLKAGKEPGLQIWRVEKF	5197.30	86.56
	1F6-Antp	MKIKIIAPPERKYSVWIGGSILASL-**RQIKIWFQNRRMKWKK**	5000.08	95.54
	Antp-1F6	**RQIKIWFQNRRMKWKK**-MKIKIIAPPERKYSVWIGGSILASL	5008.08	95.65
	Antp-1E13	**RQIKIWFQNRRMKWKK**- PHAILRLDLAGRDLTDYLMKILTER	5153.13	96.03
	1E13-Antp	PHAILRLDLAGRDLTDYLMKILTER-**RQIKIWFQNRRMKWKK**	5153.13	97.93

Table S3. Real-Time PCR primers used in the study

Gene	Primer Sequences
p53	Forward: 5' CCTCAGCATCTTATCCGAGT 3'<br>Reverse: 5' TGGATGGTGGTACAGTCAGA 3'
VDAC-1	Forward: 5' AATGACGGGACAGAGTTTGGCCA 3'<br>Reverse: 5' AGCGCGTGTTACTGTTTCCTGCCA 3'
β-Actin	Forward: 5' ACTCTTCCAGCCTTCCTTCC 3'<br>Reverse: 5' TGTTGGCGTACAGGTCTTTG 3'
GAPDH	Forward: 5' GAAGGTGAAGGTCGGAGTC 3'<br>Reverse: 5' GAAGATGGTGATGGGATTTC 3'

Table S4. List of VDAC1-interacting proteins composing the peptide array
Proteins identified as interacting with VDAC1; their functions and cellular localization are indicated. Accession codes, when available, are underlined.

No.	Protein name, MW, accession code	Function and association with VDAC1	Ref.
		Mitochondrial and apoptosis-regulating proteins	
1	**Cytochrome c,** 12.4 kDa<br>PDB-5EXQ	**Cyto** c is an electron carrier that also plays a key role in mitochondrial-mediated apoptosis. Oligomeric VDAC1 is proposed to mediate the release of Cyto c, leading to cell death. ***IMS***	[1]
2	**ANT**–ATP/ADP translocase, 32 kDa<br>AF-P12236-F1	**ANT**, ADP/ATP antiporter, mediates the import of ADP into the mitochondrial matrix for ATP synthesis, and the export of ATP out to fuel the cell. VDAC1 forms a complex with ANT and with HK, allowing coupling between OXPHOS and glycolysis. ***IMM***	[2]
3	**MAVS**–Mitochondrial antiviral-signaling protein, 57 kDa	**MAVS** was demonstrated to modulate VDAC1 protein stability via the ubiquitin–proteasome pathway, decreasing its degradative K48-linked ubiquitination. It binds to VDAC1 and induces apoptosis by caspase-3 activation, which is independent of its role in innate immunity. ***OMM***	[3, 4]
4	**CYPD**–Cyclophilin D, 42.9 kDa<br>AF-Q08752-F1	**Cyclophilin D** was found to be essential for Ca^{2+}-sensitive PTP opening. It is involved in mediating Ca^{2+}-induced cell death. ***Mitochondrial matrix***	[5]
5	**TSPO**–Translocator protein, 18 kDa<br>AF-P30536-F0,1	Human **TSPO** interacts with all three isoforms of VDAC. TSPO interaction with VDAC1 contributes to the efficiency of the mitochondrial quality control machinery**. *Mitochondrion***	[6]
6	**Bax**–Apoptosis regulator BAX isoform 1, 21.1 kDa<br>PDB_4S0O	**Bax** plays a role in the mitochondrial apoptotic process. VDAC1 and Bax form hetero oligomers forming the Cyto c release channel. Reducing VDAC1 expression by siRNA efficiently prevented cisplatin-induced apoptosis and Bax activation in NSCLC. ***Cytosol***	[7, 8]
7	**BAD, Bcl2-associated agonist of cell death,** 25.2 kDa<br>AF-O35147	**BAD** belongs to the "BH3-only proteins," a pro-apoptotic member of the Bcl-2 gene family. Upon apoptosis induction, it translocates from the cytosol to the mitochondria. Dephosphorylated BAD forms a heterodimer with anti-apoptotic proteins Bcl-2 and Bcl-xL and prevents their anti-apoptotic activity. ***Cytosol***	[9]

8	**MCL1**–Induced myeloid leukemia differentiation protein, 37.3 kDa AF-Q07820	**Mcl-1**, an anti-apoptotic protein member of the Bcl-2 family, interacts directly with VDAC1 to increase mitochondrial Ca^{2+} uptake and ROS generation, an interaction that promotes lung-cancer cell migration ***Cytosol***	[10]
		Cytoskeletal proteins	
9	**Actin**, 42 kDa PDB_5JLH	**Actin** is a highly conserved protein that polymerizes to produce filaments. It is the major protein constituent of microfilaments, which regulates a variety of cell functions such as motility, cell division, endocytosis, intracellular trafficking, chromatin remodeling, DNA repair, and regulation of transcription. Actin was shown to bind VDAC1. ***Nucleus, Cytoskeleton***	[11]
10	**TUBA1B**–Tubulin alpha-1B chain, 50.1 kDa, PDB_6EB7	**Tubulin** is a cytoskeletal protein, the major constituent of microtubules. It binds to VDAC1 and induces its closure. ***Cytoskeleton***	[12]
11	GELSOLIN, 86 kDa AF-P06396	Gelsolin is a Ca^{2+}-dependent protein that regulates actin assembly and disassembly. Human (h)Gelsolin has pro-apoptotic or anti-apoptotic activity, depending on the cell type. hGelsolin inhibits VDAC1 conductance and interacts with VDAC1 in a Ca^{2+}-dependent manner to inhibit Cyto c. hGelsolin binds and inhibits VDAC1 channel activity and Cyto c release from liposomes through direct binding to VDAC1 in a Ca^{2+}-dependent manner. ***Cytoskeletal, Cytosol***	[13, 14]
		Chaperones	
12	**GRP78**–78 kDa ER chaperone glucose-regulated protein, BiP1 precursor, AF_ P11021	BiP, also known as 78-kDa glucose-regulated protein **GRP78**, is an ATP-dependent chaperone that is ER-localized and binds directly to unfolded protein clients. No direct interaction with VDAC1 has been demonstrated. ***ER, cytosol***	[15]
13	**GRP75/HAPA9**–Stress-70 protein (HSP70), 73.9 kDa	The heat shock 70kDa protein (**HSP-70**) chaperone functions in the folding of newly synthesized proteins, translocation of polypeptides into the mitochondria, chloroplasts, and ER, and the disassembly of protein complexes. It is also known as MOT, GRP75, PBP74, HSPA9B, MTHSP75, and mortalin. VDAC1 interaction with mortalin results in modulating its channel activity and interaction with mitochondrial VDAC1, leading to mitochondria Ca^{2+} overload from the ER. ***Nucleus, Mitochondrion***	[16, 17]
		Cell signaling	
14	**GAPDH**–Glyceraldehyde -3 phosphate dehydrogenase-1, 36 kDa, PDB_1U8F	**GAPDH1** is a glycolysis enzyme, shown to regulate DNA replication, transcription, and apoptosis. It interacts with VDAC1 to modulate cellular Ca^{2+} homeostasis via interaction with the ER protein IP_3R.and acts as a cytoskeleton protein. ***Nucleus, Cytosol***	[18, 19]
15	**IκBα** -NF-kappa-B inhibitor alpha, 35.6 kDa PDB_ 1IKN	**IκBα** inhibits the activity of dimeric NF-kappa-B/REL complexes by masking its NLS, thus, keeping it in an inactive state in the cytoplasm. IkBα phosphorylation, ubiquitination, and degradation by the proteasome leads to activation of NF-kB. ***Nucleus, Cytosol***	**[20]**
16	**CDK2**–Cyclin-dependent kinase 2, 33.9 kDa, PDB_1AQ1	**CDK2** is a serine/threonine-protein kinase that controls the cell cycle and is essential for meiosis, but dispensable for mitosis. ***Nucleus, Cytosol, Centrosome***	[21]
17	**CD4**–T-cell surface glycoprotein (isoform 1), 51.1 kDa	**CD4** is a membrane glycoprotein and co-receptor of the T-cell receptor (TCR). It assists TCR in communicating with antigen-presenting cells, and binds to MHC class II-restricted T-cell activation. It is involved in T-cell antigen recognition and activation. No direct interaction with VDAC1 has been reported. ***Plasma membrane***	[22]
18	**SNCA**–α-synuclein, 14.4 kDa	**α-synuclein**, implicated in Parkinson's disease, binds to VDAC and modifies its properties towards higher Ca^{2+} permeability. VDAC could be one of the pathways for α-synuclein translocation across the OMM. ***Nucleus, Cytosol***	[23, 24]

19	**SOD1**–Superoxide dismutase, 15.9 kDa	**SOD**1 mutated protein is associated with ALS. It interacts with VDAC1, reducing its channel conductance and altering the interaction between VDAC1 and Bcl-2. ***Cytosol, Mitochondrion***	[25]

Table S5. Peptides derived from VDAC1-interacting proteins as identified following incubation with VDAC1 and detection by anti-VDAC1 against internal or N-terminal domain sequences
The detected proteins, the spots in the array interacted with VDAC1, and the sequence of the peptide are shown. VDAC1 interaction with the peptides was detected by antibodies against VDAC1 internal sequence (amino acids 150-250) or the N-terminal domain sequences. +, ++ or +++ present spot intensity: low, medium and high, respectively. The results presented as 3 groups: sequence detected by both antibodies, or only by antibodies against VDAC1 internal sequence or N-terminal domain.

No.	Protein name	Spot	Sequence	Detected using anti-VDAC1 internal sequence	Detected using anti-N-terminus VDAC1
**Peptides detected by both antibodies**					
1.	**Apoptosis regulator isoform 1 (BAX)**	1B12	RERLLGWIQDQGGWGLPLAESLKRL	+	+++
2.		1B13	DQGGWGLPLAESLKRLMSLSPGRPP	+	+++
3.	**Alpha-synuclein isoform**	1C2	EGVVHGVATVAEKTKEQVTNVGGAV	+	++
4.	**Cytochrome c**	1D15	IFVGIKKKEERADLIAYLKKATNE	+	++
5.		1D16	IKKKEERADLIAYLKKATNE	+	++
6.	**Actin, cytoplasmic 1**	1E13	PHAILRLDLAGRDLTDYLMKILTER	+	++
7.		1E14	AGRDLTDYLMKILTERGYSFTTTAE	+	++
8.	**ADP/ATP translocase 3 (ANT3)**	1F19	EQGVLSFWRGNLANVIRYFPTQALN	+	+++
9.		1F20	GNLANVIRYFPTQALNFAFKDKYKQ	+	+++
10.	**T-cell surface glycoprotein CD4 isoform 1 precursor**	1M5	NSNQIKILGNQGSFLTKGPSKLNDR	+	+++
11.	**Cyclin-dependent kinase 2 (CDK2)**	1O7	LDTETEGVPSTAIREISLLKELNHP	+	+++
12.		1O8	STAIREISLLKELNHPNIVKLLDVI	+++	+++
13.		1O13	MDASALTGIPLPLIKSYLFQLLQGL	+	+++
14.		1P1	RRALFPGDSEIDQLFRIFRTLGTPD	++	++
15.		1P2	EIDQLFRIFRTLGTPDEVVWPGVTS	++	+
16.	**Tubulin, alpha 1b**	2A3	LVFHSFGGGTGSGFTSLLMERLSVD	+	+++
17.		2A4	TGSGFTSLLMERLSVDYGKKSKLEF	+	+
18.		2A11	AIYDICRRNLDIERPTYTNLNRLIS	+	+++
19.		2A14	VSSITASLRFDGALNVDLTEFQTNL	+++	+
20.	**GAPDH1**	2C6	KIISNASCTTNCLAPLAKVIHDNFG	++	+++
21.		2C18	AKYDDIKKVVKQASEGPLKGILGYT	++	+++
22.	**NF-kappa-B inhibitor alpha**	2F17	ASIHGYLGIVELLVSLGADVNAQEP	+	++
23.	**ER chaperone BiP1 precursor**	2K14	RQATKDAGTIAGLNVMRIINEPTAA	++	++
24.		2K15	IAGLNVMRIINEPTAAAIAYGLDKR	++	++
25.		2K20	LTIDNGVFEVVATNGDTHLGGEDFD	+	+

26.		2L4	IEIESFYEGEDFSETLTRAKFEELN	++	+
27.		2L10	LVGGSTRIPKIQQLVKEFFNGKEPS	+++	+++
28.		2L11	KIQQLVKEFFNGKEPSRGINPDEAV	+++	+++
29.		2L22	ERPLTKDNHLLGTFDLTGIPPAPRG	+	++
30.		2L23	LLGTFDLTGIPPAPRGVPQIEVTFE	+	+++
31.		2M14	IEDFKAKKKELEEIVQPIISKLYGS	++	++
32.		2M15	ELEEIVQPIISKLYGSAGPPPTGEE	+++	++
33.	**Stress-70 protein, mitochondrial precursor (HSP-70)**	2N23	SILEIQKGVFEVKSTNGDTFLGGED	+++	+++
34.		2O9	NMKLTRAQFEGIVTDLIRRTIAPCQ	++	+
35.		2O19	TDVLLLDVTPLSLGIETLGGVFTKL	+	++
36.		2O20	PLSLGIETLGGVFTKLINRNTTIPT	++	++
**Peptides detected by antibodies against VDAC1 internal sequence**					
1.	**Superoxide dismutase [Cu-Zn] (SOD1)**	1C16	GPVKVWGSIKGLTEGLHGFHVHEFG	+++	-
2.		1C17	KGLTEGLHGFHVHEFGDNTAGCTSA	+++	-
3.	**Translocator protein (TSPO)**	1H10	AAAATTVAWYQVSPLAARLLYPYLA	++	-
4.		1H11	YQVSPLAARLLYPYLAWLAFTTTLN	+	-
5.	**Mitochondrial antiviral-signaling protein isoform 1 (MAVS)**	1L13	YKSEGTFGIHVAENPSIQLLEGNPG	++	-
6.		1L14	HVAENPSIQLLEGNPGPPADPDGGP	+++	-
7.	**Tubulin, alpha 1b**	2A12	LDIERPTYTNLNRLISQIVSSITAS	++	-
8.		2A15	FDGALNVDLTEFQTNLVPYPRIHFP	++	-
9.	**GAPDH1**	2B20	KFHGTVKAENGKLVINGNPITIFQE	+++	-
10.		2C23	GAGIALNDHFVKLISWYDNEFGYSN	+	-
11.		2C24	FVKLISWYDNEFGYSNRVVDLMAHM	+	-
12.		2D1	NEFGYSNRVVDLMAHMASKE	+++	-
13.	**Induced myeloid leukemia cell differentiation protein Mcl-1 isoform 1**	2D23	DELYRQSLEIISRYLREQATGAKDT	+	-
14.	**NF-kappa-B inhibitor alpha**	2G2	QLTLENLQMLPESEDEESYDTESEF	++	-
15.	**Gelsolin isoform b**	2I10	EVAASAILTAQLDEELGGTPVQSRV	+	-
**Peptides detected by antibodies against the VDAC1-N-Terminus**					
1.	**Bcl-2 associated agonist of cell death**	1A9	HSSYPAGTEDDEGMGEEPSPFRGRS	-	++
2.		1A10	DDEGMGEEPSPFRGRSRSAPPNLWA	-	++
3.		1A11	SPFRGRSRSAPPNLWAAQRYGRELR	-	+
4.		1A13	RYGRELRRMSDEFVDSFKKGLPRPK	-	+
5.		1A15	KGLPRPKSAGTATQMRQSSSWTRVF	-	++
6.		1A16	GTATQMRQSSSWTRVFQSWWDRNLG	-	++
7.		1A17	SSWTRVFQSWWDRNLGRGSSAPSQ	-	+
8.	**Apoptosis regulator BAX isoform 1**	1A21	GGGPTSSEQIMKTGALLLQGFIQDR	-	+
9.		1A22	IMKTGALLLQGFIQDRAGRMGGEAP	-	+++
10.		1B5	AVDTDSPREVFFRVAADMFSDGNFN	-	+++

11.		1B8	RVVALFYFASKLVLKALCTKVPELI	-	++
12.		1B9	SKLVLKALCTKVPELIRTIMGWTLD	-	+
13.		1B10	TKVPELIRTIMGWTLDFLRERLLGW	-	+++
14.		1B11	IMGWTLDFLRERLLGWIQDQGGWGL	-	+++
15.	**Alpha-synuclein isoform NACP140**	1C4	TNVGGAVVTGVTAVAQKTVEGAGSI	-	+
16.		1C5	GVTAVAQKTVEGAGSIAAATGFVKK	-	++
17.	**SOD1**	1C18	FHVHEFGDNTAGCTSAGPHFNPLSR	-	++
18.	**Cytochrome c**	1D8	HTVEKGGKHKTGPNLHGLFGRKTGQ	-	+
19.		1D9	KTGPNLHGLFGRKTGQAPGYSYTAA	-	+
20.	**Actin, cytoplasmic 1**	1D20	AGFAGDDAPRAVFPSIVGRPRHQGV	-	+++
21.		1E15	MKILTERGYSFTTTAEREIVRDIKE	-	+
22.		1F5	EITALAPSTMKIKIIAPPERKYSVW	-	++
23.		1F6	MKIKIIAPPERKYSVWIGGSILASL	-	+++
24.	**ADP/ATP translocase 3**	1F12	MTEQAISFAKDFLAGGIAAAISKTA	-	+
25.		1F13	KDFLAGGIAAAISKTAVAPIERVKL	-	++
26.		1F18	VDCIVRIPKEQGVLSFWRGNLANVI	-	++
27.		1F21	FPTQALNFAFKDKYKQIFLGGVDKH	-	+++
28.		1F22	FKDKYKQIFLGGVDKHTQFWRYFAG	-	+
29.		1G4	VGKSGTEREFRGLGDCLVKITKSDG	-	+++
30.		1G5	FRGLGDCLVKITKSDGIRGLYQGFS	-	+++
31.		1G17	FRDEGGKAFFKGAWSNVLRGMGGAF	-	+
32.	**Peptidylprolyl isomerase D (Cyclophilin D)**	1I7	TVPTPHLDGKHVVFGQVIKGIGVAR	-	+
33.	**MAVS1**	1K9	AGATSSLTPSRGPVSPSVSFQPLAR	-	+++
34.	**T cell surface glycoprotein CD4 isoform 1 precursor**	1M4	KKSIQFHWKNSNQIKILGNQGSFLT	-	+
35.		1M6	NQGSFLTKGPSKLNDRADSRRSLWD	-	++
36.		1M7	PSKLNDRADSRRSLWDQGNFPLIIK	-	++
37.		1N4	SVKRVTQDPKLQMGKKLPLHLTLPQ	-	++
38.		1N5	KLQMGKKLPLHLTLPQALPQYAGSG	-	++
39.	**Tubulin, alpha 1b**	2A2	DQCTGLQGFLVFHSFGGGTGSGFTS	-	+
40.		2B2	FVDWCPTGFKVGINYQPPTVVPGGD	-	+
41.		2B5	KVQRAVCMLSNTTAIAEAWARLDHK	-	+
42.		2B6	SNTTAIAEAWARLDHKFDLMYAKRA	-	++
43.	**GAPDH isoform 1**	2B14	MGKVKVGVNGFGRIGRLVTRAAFNS	-	+++
44.		2B21	NGKLVINGNPITIFQERDPSKIKWG	-	+
45.		2C5	NHEKYDNSLKIISNASCTTNCLAPL	-	+
46.		2C10	ITATQKTVDGPSGKLWRDGRGALQN	-	+
47.		2C13	PASTGAAKAVGKVIPELNGKLTGMA	-	+
48.		2C14	VGKVIPELNGKLTGMAFRVPTANVS	-	+++
49.	**Induced myeloid leukemia cell differentiation**	2D10	AVIGGSAGASPPSTLTPDSRRVARP	-	+
50.		2D11	SPPSTLTPDSRRVARPPPIGAEVPD	-	+

51.	**protein Mcl-1 isoform 1**	2D22	TPPPAEEEEDELYRQSLEIISRYLR	-	++
52.		2E5	TAFQGMLRKLDIKNEDDVKSLSRVM	-	+
53.		2E12	PLAESITDVLVRTKRDWLVKQRGWD	-	++
54.	**NF kappa B inhibitor alpha**	2F6	MEVIRQVKGDLAFLNFQNNLQQTPL	-	+
55.		2F13	QGCLASVGVLTQSCTTPHLHSILKA	-	++
56.		2F24	PYQLTWGRPSTRIQQQLGQLTLENL	-	+++
57.		2G1	STRIQQQLGQLTLENLQMLPESEDE	-	++
58.		2G5	FTEDELPYDDCVFGGQRLTL	-	+
59.		2G6	TEDELPYDDCVFGGQRLTL	-	+
60.	**Gelsolin isoform b**	2G8	MVVEHPEFLKAGKEPGLQIWRVEKF	-	+++
61.		2G18	RAVQHREVQGFESATFLGYFKSGLK	-	+
62.		2H20	QVSVLPEGGETPLFKQFFKNWRDPD	-	++
63.		2I12	TPVQSRVVQGKEPAHLMSLFGGKPM	-	+
64.		2I13	GKEPAHLMSLFGGKPMIIYKGGTSR	-	+
65.		2I15	YKGGTSREGGQTAPASTRLFQVRAN	-	+
66.	**Endoplasmic reticulum chaperone BiP1 precursor** (GRP78)	2K5	GRTWNDPSVQQDIKFLPFKVVEKKT	-	+
67.		2K10	ISAMVLTKMKETAEAYLGKKVTHAV	-	++
68.		2L3	ALSSQHQARIEIESFYEGEDFSETL	-	++
69.		2L5	EDFSETLTRAKFEELNMDLFRSTMK	-	+
70.		2L17	LGIETVGGVMTKLIPRNTVVPTKKS	-	++
71.	**Stress-70 protein, mitochondrial precursor (HSP-70)**	2M21	RGPTAARHQDSWNGLSHEAFRLVSR	-	+
72.		2O6	KCELSSSVQTDINLPYLTMDSSGPK	-	+
73.		2O21	GGVFTKLINRNTTIPTKKSQVFSTA	-	+
74.		2P3	LIGIPPAPRGVPQIEVTFDIDANGI	-	+

Table S6. Peptides derived from VDAC1-interacting proteins as identified by anti-VDAC1 antibodies against internal sequence or N-terminal domain

The VDAC1-interacting sequences derived from the indicated proteins and represented by the indicated spot number in the array were detected using antibodies against the VDAC1-N- terminus or VDAC1 internal sequences. Representative spots as labeled by squares or circles in Fig. 1C and D are presented with "+" indicating their detection with the indicated antibody.

No.	Protein name	Spot	Sequence	Detected with anti-VDAC1 against:	
				Internal sequence	N-terminus
1	Superoxide dismutase [Cu-Zn] (SOD1)	1C16	GPVKVWGSIKGLTEGLHGFHVHEFG	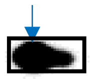	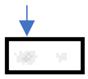
2		1C17	KGLTEGLHGFHVHEFGDNTAGCTSA	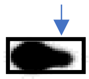	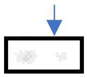
3	GAPDH1	2D1	NEFGYSNRVVDLMAHMASKE	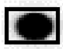	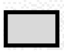
4		2C23	GAGIALNDHFVKLISWYDNEFGYSN	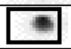	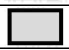

**5**	**Actin**	1F5	EITALAPSTMKIKIIAPPERKYSVW	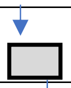	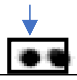
**6**		1F6	MKIKIIAPPERKYSVWIGGSILASL	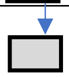	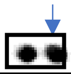
**7**<br>**8**	**Gelsolin isoform b**	2G8	MVVEHPEFLKAGKEPGLQIWRVEKF	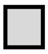	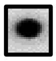
**9**		2I13	GKEPAHLMSLFGGKPMIIYKGGTSR	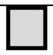	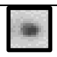
**10**		2I15	YKGGTSREGGQTAPASTRLFQVRAN	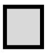	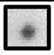

Table S7. Effect of the VDAC1-interacting peptides derived from its partners on cell viability and cell death

Summary of the effects of VDAC1-interacting CPP peptides on cell death and cell viability. The cell penetrating sequences are blue and the targeted compartment is indicated. The concentrations required for 50% inhibtion of cell survival or induction of cell death (IC_{50}) are indicated. Results represent mean values ± SE (n = 3).

**Protein**	**Peptide and targeting**	**Cell viability, U87-MG**		**Cell death**			
				**U87-MG**		**PC-3**	
		**Maximal inhibition, %**	**IC_{50} , µM**	**Maximal, %**	**IC_{50} , µM**	**Maximal, %**	**IC_{50} , µM**
**GAPDH1**	2D1-Nuc<br>2D1-Antp	0<br>75	>>25<br>10	10<br>100	>>25<br>8	5<br>80	>>25<br>14
**Gelsolin isoform b**	2G8-Antp<br>Antp-2G8	50<br>30	>100<br>>100	100<br>0	10<br>>>100	100<br>80	5<br>68
**Actin**	1F6 Antp<br>Antp-1F6<br>Antp-1E13<br>1E13-Antp	-<br>-<br>-<br>-	-<br>-<br>-<br>-	100<br>100<br>100<br>100	8<br>8<br>8<br>8	97<br>97<br>87<br>87	16<br>10<br>10<br>10

Actin

VDNGSGMCKAGFAGDDAPRAVFPSI	1D19	
AGFAGDDAPRAVFPSIVGRPRHQGV	1D20	*
RAVFPSIVGRPRHQGVMVGMGQKDS	1D21	
VPIYEGYALPHAILRLDLAGRDLTD	1E12	
PHAILRLDLAGRDLTDYLMKILTER	1E13	***
AGRDLTDYLMKILTERGYSFTTTAE	1E14	***
MKILTERGYSFTTTAEREIVRDIKE	1E15	*
SFTTTAEREIVRDIKEKLCYVALDF	1E16	
PGIADRMQKEITALAPSTMKIKIIA	1F4	
EITALAPSTMKIKIIAPPERKYSVW	1F5	*
MKIKIIAPPERKYSVWIGGSILASL	1F6	*
ERKYSVWIGGSILASLSTFQQMWIS	1F7	

Gelsolin isoform b

MVVEHPEFLKAGKEPGLQIWRVEKF	2G8	*
KAGKEPGLQIWRVEKFDLVPVPTNL	2G9	
EVAASAILTAQLDEELGGTPVQSRV	2I10	**
AQLDEELGGTPVQSRVVQGKEPAHL	2I11	
TPVQSRVVQGKEPAHLMSLFGGKPM	2I12	*
GKEPAHLMSLFGGKPMIIYKGGTSR	2I13	*
LFGGKPMIIYKGGTSREGGQTAPAS	2I14	
YKGGTSREGGQTAPASTRLFQVRAN	2I15	*
GQTAPASTRLFQVRANSAGATRAVE	2I16	

GAPDH 1

MGKVKVGVNGFGRIGRLVTRAAFNS	2B14	*
GFGRIGRLVTRAAFNSGKVDIVAIN	2B15	
MFQYDSTHGKFHGTVKAENGKLVIN	2B19	
KFHGTVKAENGKLVINGNPITIFQE	2B20	**
NGKLVINGNPITIFQERDPSKIKWG	2B21	*
PITIFQERDPSKIKWGDAGAEYVVE	2B22	
DAPMFVMGVNHEKYDNSLKIISNAS	2C4	
NHEKYDNSLKIISNASCTTNCLAPL	2C5	*
KIISNASCTTNCLAPLAKVIHDNFG	2C6	***
TNCLAPLAKVIHDNFGIVEGLMTTV	2C7	
EGLMTTVHAITATQKTVDGPSGKLW	2C9	
ITATQKTVDGPSGKLWRDGRGALQN	2C10	*
GPSGKLWRDGRGALQNIIPASTGAA	2C11	
DLTCRLEKPAKYDDIKKVVKQASEG	2C17	
AKYDDIKKVVKQASEGPLKGILGYT	2C18	***
VKQASEGPLKGILGYTEHQVVSSDF	2C19	
DTHSSTFDAGAGIALNDHFVKLISW	2D22	
GAGIALNDHFVKLISWYDNEFGYSN	2C23	**
FVKLISWYDNEFGYSNRVVDLMAHM	2C24	**
NEFGYSNRVVDLMAHMASKE	2D1	**
FGYSNRVVDLMAHMASKE	2D2	

Fig. S1. Peptide sequences directly interact with VDAC1 as identified by antibodies against VDAC1 internal sequences or the N-terminus domain.

The sequence of the VDAC1-interacting peptides arranges in sub-groups, their protein of origin, and spot number in the peptide array are indicated. The interacting peptide is in red, peptides located before and after it are in black, and sequences in blue showed weak interaction. The different colors highlight sequences and indicate the color presenting the specific peptide in the 3D-structure.

Interaction with antibody against the N-terminus, or the internal sequence or with both are presented as *, **,***, respectively

1. Actin, cytoplasmic 1 [Homo sapiens]

NCBI Reference Sequence: NP_001092.1]

**MDDDIAALVVDNGSGMCKAGFAGDDAPRAVFPSIVGRPRHQGVMVGMGQKDSYVGDEAQSKRGILTLKYPIEHGIVTN
WDDMEKIWHHTFYNELRVAPEEHPVLLTEAPLNPKANREKMTQIMFETFNTPAMYVAIQAVLSLYASGRTTGIVMDSG
DGVTHTVPIYEGYALPHAILRLDLAGRDLTDYLMKILTERGYSFTTTAEREIVRDIKEKLCYVALDFEQEMATAASSSLE
KSYELPDGQVITIGNERFRCPEALFQPSFLGMESCGIHETTFNSIMKCDVDIRKDLYANTVLSGGTTMYPGIADRMQKEI
TALAPSTMKIKIIAPPERKYSVWIGGSILASLSTFQQMWISKQEYDESGPSIVHRKCF**

2. Gelsolin isoform b [Homo sapiens]

NCBI Reference Sequence: NP_001339982.1

**MVVEHPEFLKAGKEPGLQIWRVEKFDLVPVPTNLYGDFFTGDAYVILKTVQLRNGNLQYDLHYWLGNECSQDESGAAAI
FTVQLDDYLNGRAVQHREVQGFESATFLGYFKSGLKYKKGGVASGFKHVVPNEVVVQRLFQVKGRRVVRATEVPVSWES
FNNGDCFILDLGNNIHQWCGSNSNRYERLKATQVSKGIRDNERSGRARVHVSEEGTEPEAMLQVLGPKPALPAGTEDTAK
EDAANRKLAKLYKVSNGAGTMSVSLVADENPFAQGALKSEDCFILDHGKDGKIFVWKGKQANTEERKAALKTASDFITK
MDYPKQTQVSVLPEGGETPLFKQFFKNWRDPDQTDGLGLSYLSSHIANVERVPFDAATLHTSTAMAAQHGMDDDGTGQ
KQIWRIEGSNKVPVDPATYGQFYGGDSYIILYNYRHGGRQGQIIYNWQGAQSTQDEVAASAILTAQLDEELGGTPVQSRVV
QGKEPAHLMSLFGGKPMIIYKGGTSREGGQTAPASTRLFQVRANSAGATRAVEVLPKAGALNSNDAFVLKTPSAAYLWVG
TGASEAEKTGAQELLRVLRAQPVQVAEGSEPDGFWEALGGKAAYRTSPRLKDKKMDAHPPRLFACSNKIGRFVIEEVPGE
LMQEDLATDDVMLLDTWDQVFVWVGKDSQEEEKTEALTSAKRYIETDPANRDRRTPITVVKQGFEPPSFVGWFLGWDD
DYWSVDPLDRAMAELAA**

3. Glyceraldehyde-3-phosphate dehydrogenase isoform 1 [Homo sapiens]

NCBI Reference Sequence: NP_001276675.1

**MGKVKVGVNGFGRIGRLVTRAAFNSGKVDIVAINDPFIDLNYMVYMFQYDSTHGKFHGTVKAENGKLVINGNPITIFQER
DPSKIKWGDAGAEYVVESTGVFTTMEKAGAHLQGGAKRVIISAPSADAPMFVMGVNHEKYDNSLKIISNASCTTNCLAPL
AKVIHDNFGIVEGLMTTVHAITATQKTVDGPSGKLWRDGRGALQNIIPASTGAAKAVGKVIPELNGKLTGMAFRVPTANVS
VVDLTCRLEKPAKYDDIKKVVKQASEGPLKGILGYTEHQVVSSDFNSDTHSSTFDAGAGIALNDHFVKLISWYDNEFGYSN
RVVDLMAHMASKE**

Fig. S2. VDAC1-interacting sequences in VDAC1-interacting protein sequences

Protein sequences of the proteins from homo sapiens addressed in this study presented with the VDAC1-interacting sequences are in red. The highlighted sequences indicate those used as CPP

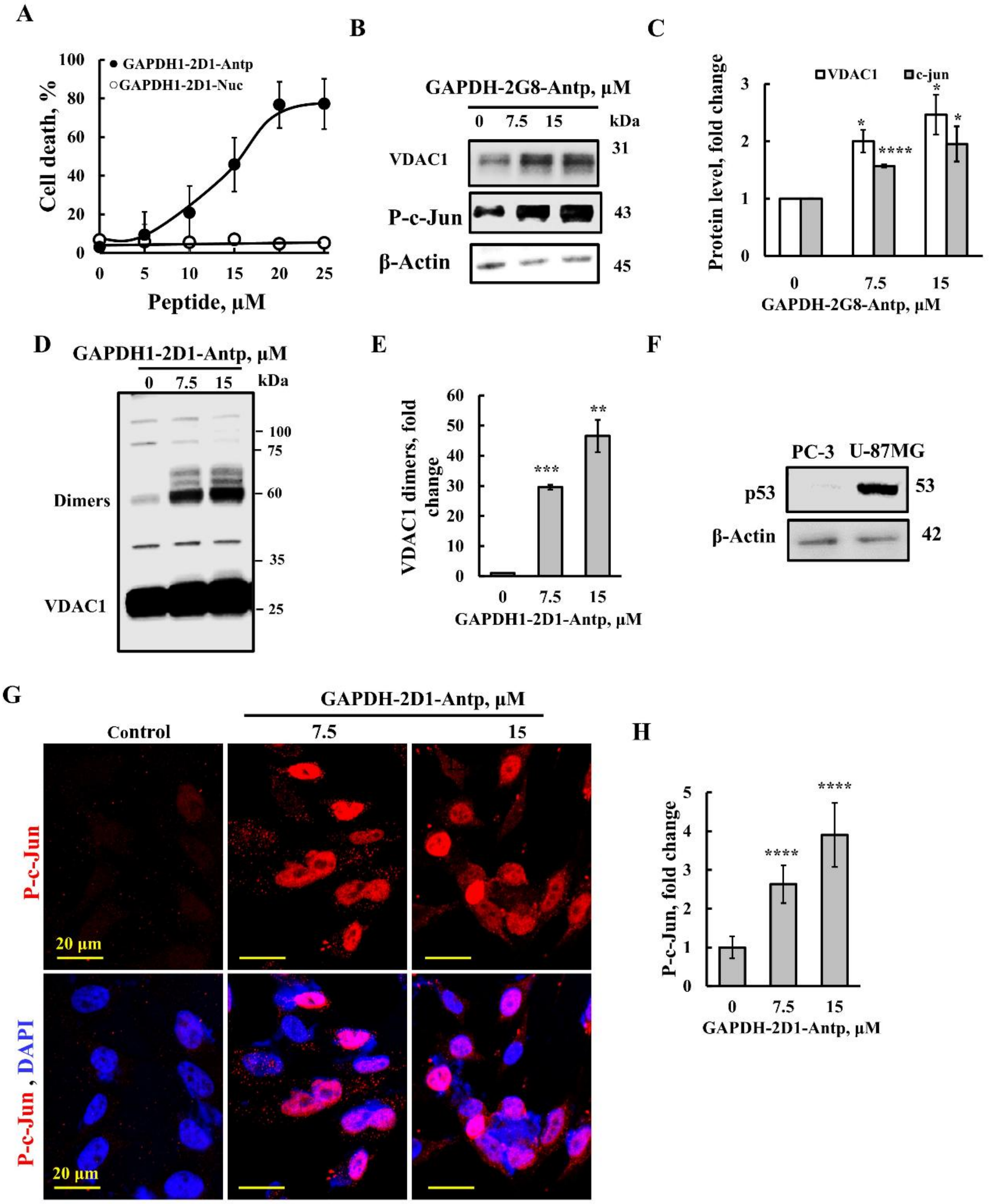

Fig. S3. VDAC1-interacting, GAPDH-derived peptide induced apoptosis, VDAC1 expression its oligomerization, and increased P-c-Jun levels in p53- lucking PC-3 cells

(A) PC-3 cells were incubated for 24 h with the indicated concentrations (0-25 µM) of cytosol-targeted peptide GAPDH1-2D1-Antp or nucleus targeted peptide GAPDH1-2D1-Nuc in a serum-free medium followed by analysis of cell death using PI staining and flow cytometry analysis. **(B,C)** Cells (200,000 cells/well, 6 well plate) were incubated for 24 h with 7.5 or 15 µM of GAPDH1-2D1-Antp peptide in a serum-free medium then subjected to immunoblotting for VDAC1, P-c-Jun, or β-actin **(B)**, and bands intensity were quantified using ImageJ software **(C)**. **(D,E)** PC-3 cells were treated with 7.5 or 15 µM peptide as above, harvested and subjected to cross-linking using EGS (100 µM, 1 mg protein/ml) and immunoblotting to monitor VDAC1 oligomerization **(D)** and VDAC1 dimers were quantified **(E).** (**F**) U-87MG and PC-3 cells were analyzed for p53 expression by immunoblotting using anti-p53 antibodies. **(G,H)** PC-3 cells (100,000 cells/well, 12 well plate) seeded on coverslips were treated with 5 or 15 µM peptide and then IF stained for P-c-Jun using specific antibodies and nucleus with DAPI (blue) **(G)** and staining intensity was quantified **(H)**. Results represent the means ± SEM (n = 3), **p<0.01; ***p ≤ 0.001; ****p ≤ 0.0001.

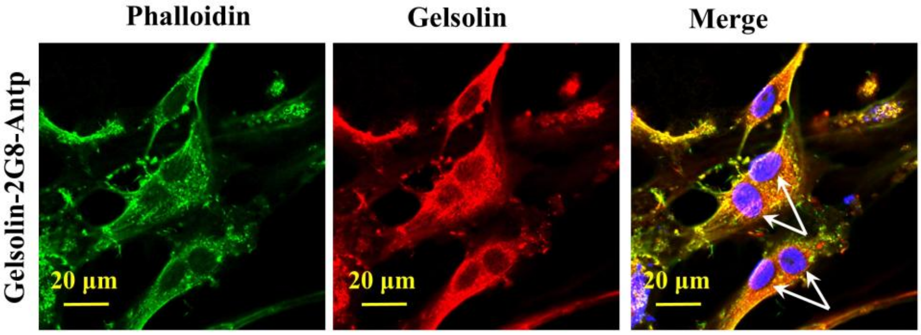

Fig. S4. VDAC1-interacting, gelsolin-derived peptide altered cell division

U-87MG cells (100,000 cells/well, 12 well plate) seeded on coverslips were treated for 24h with 15 µM of Gel-2G8-Antp peptide then stained for actin using Phalloidin-488 and for gelsolin using specific antibodies and nucleus with DAPI (blue) and visualized by confocal microscopy. Arrows point to cells with 2 nuclei.

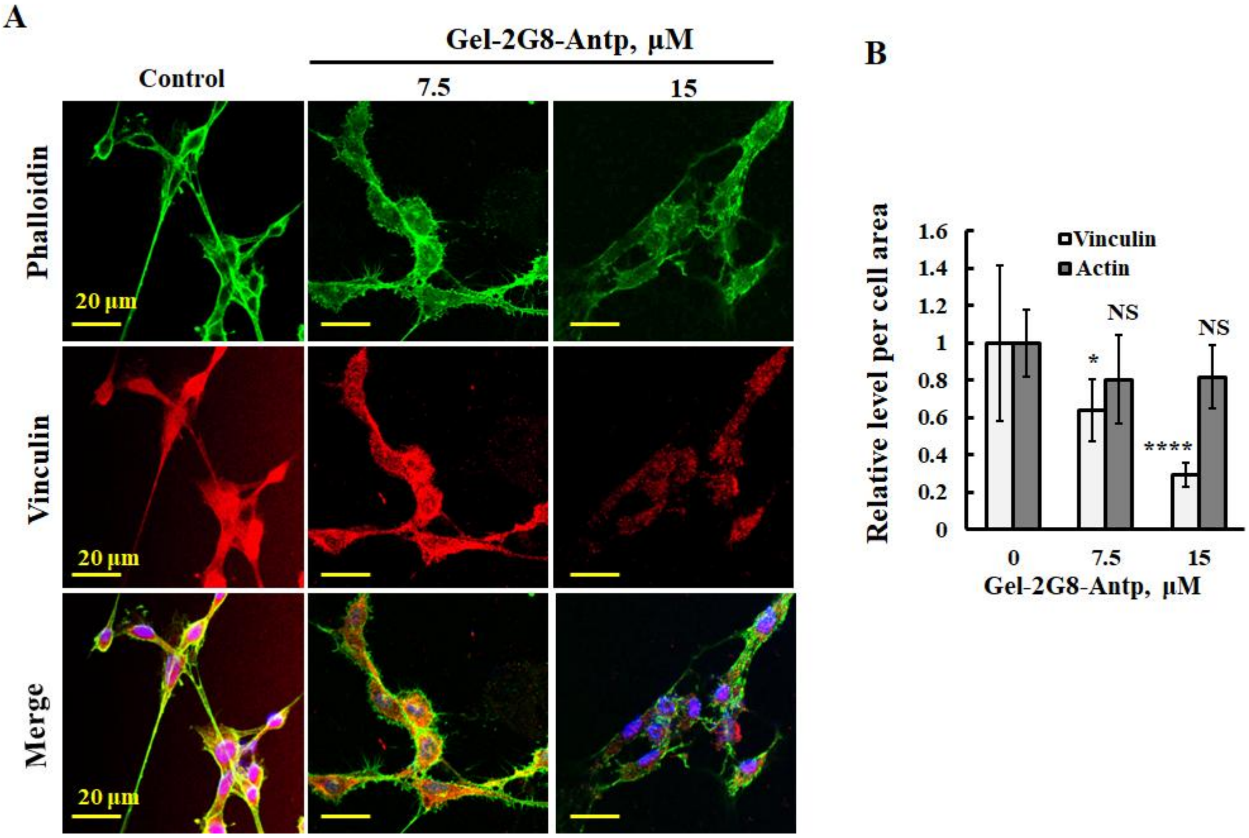

Fig. S5. VDAC1-interacting, gelsolin-derived peptide decreasing vinculin expression

(A,B) U-87MG cells (100,000 cells/well, 12 well plate) seeded on coverslips were treated for 24h with 7.5 or 15 µM of Gel-2G8-Antp peptide then stained for actin using Phalloidin-488 and vinculin using specific antibodies and nucleus with DAPI (blue) and visualized by confocal microscopy **(A)**. Staining intensity was quantified **(B)**. Results represent the means ± SEM (n = 3), *$p<0.05$; ****$p \leq 0.0001$, NS, non-specific.

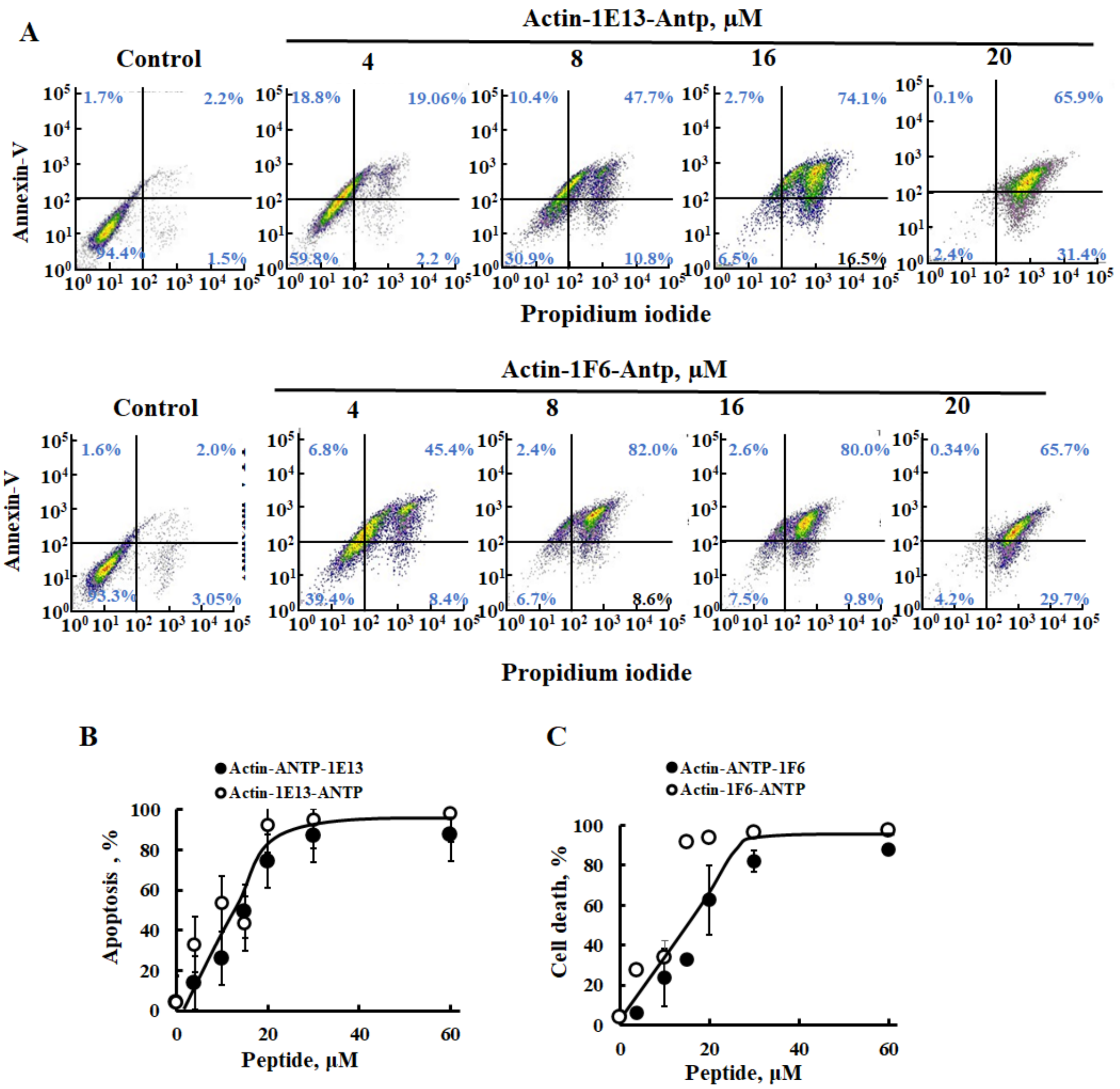

Fig. S6. VDAC1-interacting, actin-derived peptide induced apoptosis

PC-3 cells (200,000 cells/well, 6 well plate) were incubated for 24h with the indicated concentration (0-20 µM) of actin-1E13−Antp, actin-Antp−1E13, actin-Antp-1F6 or actin-1F6-Antp peptide in a serum-free medium, then harvested, and analysis for apoptosis using FITC–annexin V/PI staining and flow cytometry analysis **(A,B)** or analyzed for cell death using PI staning **(C)**. Results represent the means ± SEM (n = 3)

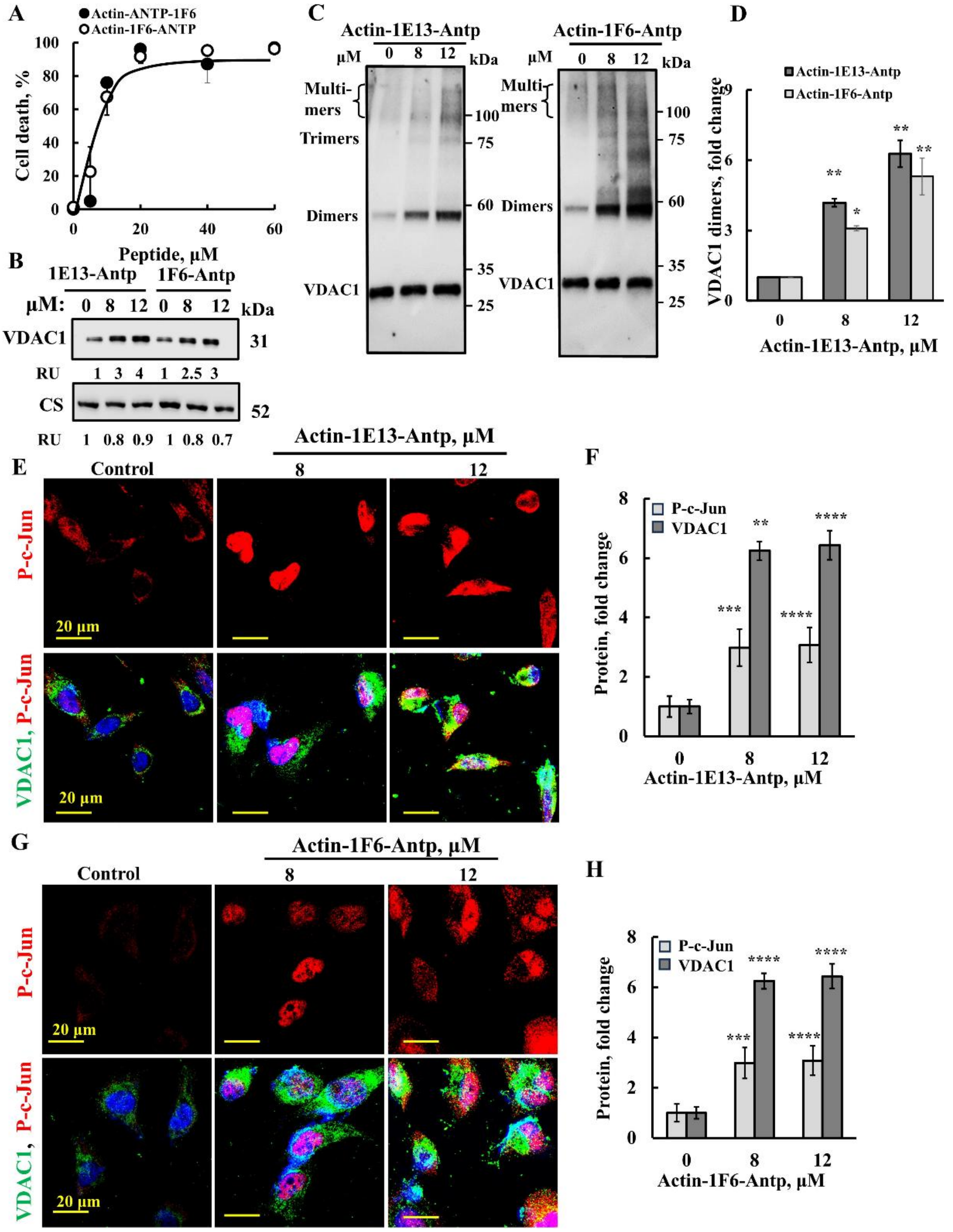

Fig. S7. VDAC1-interacting, actin-derived peptide induced apoptosis, VDAC1 expression, and its oligomerization, and enhanced P-c-Jun levels in PC-3 cells

(A-C) PC-3 cells (200,000 cells/well, 6 well plate) were incubated for 24 h in a serum-free medium with the indicated concentrations (0-60 µM) of cytosol-targeted peptide actin-1E13–Antp or actin-1F6-Antp in a serum-free medium followed by analysis of cell death using PI staining and flow cytometry analysis **(A)**, or subjected to immunoblotting for VDAC1 and citrate synthase (CS), and bands intensity were quantified using ImageJ software and presented as RU **(B)**. **(C,D)**. PC-3 cells were treated with 8 or 12 µM peptide as above, harvested and subjected to cross-linking using EGS (100 µM, 1 mg protein/ml) and immunoblotting to monitor VDAC1 oligomerization **(C)** and VDAC1 dimers were quantified **(D). (E-H)** PC-3 cells (100,000 cells/well, 12 well plate) seeded on coverslips were treated (8 or 12 µM) with actin-1E13–Antp **(E,F)** or actin-1F6-Antp peptides **(G,H)** as above and IF stained for P-c-Jun and VDAC1 using specific antibodies and nucleus with DAPI (blue) **(E,G)** and staining intensity was quantified **(F,H)**. Results represent the means ± SEM (n = 3), $^{**}p<0.01$; $^{***}p \leq 0.001$; $^{****}p \leq 0.0001$.

References

1. Shoshan-Barmatz, V., E.N. Maldonado, and Y. Krelin, *VDAC1 at the crossroads of cell metabolism, apoptosis and cell stress.* Cell Stress, 2017. **1**(1): p. 11-36.
2. Vyssokikh, M., et al., *The intra-mitochondrial cytochrome c distribution varies correlated to the formation of a complex between VDAC and the adenine nucleotide translocase: this affects Bax-dependent cytochrome c release.* Biochim Biophys Acta, 2004. **1644**(1): p. 27-36.
3. Guan, K., et al., *MAVS regulates apoptotic cell death by decreasing K48-linked ubiquitination of voltage-dependent anion channel 1.* Molecular and cellular biology, 2013. **33**(16): p. 3137-3149.
4. Trishna, S., et al., *Overexpression of the mitochondrial anti-viral signaling protein, MAVS, in cancers is associated with cell survival and inflammation.* Mol Ther Nucleic Acids, 2023. **33**: p. 713-732.
5. Crompton, M., S. Virji, and J.M. Ward, *Cyclophilin-D binds strongly to complexes of the voltage-dependent anion channel and the adenine nucleotide translocase to form the permeability transition pore.* European Journal of Biochemistry, 1998. **258**(2): p. 729-735.
6. Shoshan-Barmatz, V., S. Pittala, and D. Mizrachi, *VDAC1 and the TSPO: expression, interactions, and associated functions in health and disease states.* International journal of molecular sciences, 2019. **20**(13): p. 3348.
7. Banerjee, J. and S. Ghosh, *Bax increases the pore size of rat brain mitochondrial voltage-dependent anion channel in the presence of tBid.* Biochem Biophys Res Commun, 2004. **323**(1): p. 310-4.
8. Tajeddine, N., et al., *Hierarchical involvement of Bak, VDAC1 and Bax in cisplatin-induced cell death.* Oncogene, 2008. **27**(30): p. 4221-32.
9. Yang, E., et al., *Bad, a heterodimeric partner for Bcl-XL and Bcl-2, displaces Bax and promotes cell death.* Cell, 1995. **80**(2): p. 285-291.
10. Huang, H., et al., *Mcl-1 promotes lung cancer cell migration by directly interacting with VDAC to increase mitochondrial Ca 2+ uptake and reactive oxygen species generation.* Cell death & disease, 2014. **5**(10): p. e1482-e1482.
11. Magri, A., S. Reina, and V. De Pinto, *VDAC1 as Pharmacological Target in Cancer and Neurodegeneration: Focus on Its Role in Apoptosis.* Front Chem, 2018. **6**: p. 108.
12. Rostovtseva, T.K. and S.M. Bezrukov, *VDAC inhibition by tubulin and its physiological implications.* Biochim Biophys Acta, 2012. **1818**(6): p. 1526-35.
13. Qiao, H. and J.R. McMillan, *Gelsolin segment 5 inhibits HIV-induced T-cell apoptosis via Vpr-binding to VDAC.* FEBS letters, 2007. **581**(3): p. 535-540.
14. Kusano, H., et al., *Human gelsolin prevents apoptosis by inhibiting apoptotic mitochondrial changes via closing VDAC.* Oncogene, 2000. **19**(42): p. 4807-14.
15. Pobre, K.F.R., G.J. Poet, and L.M. Hendershot, *The endoplasmic reticulum (ER) chaperone BiP is a master regulator of ER functions: Getting by with a little help from ERdj friends.* J Biol Chem, 2019. **294**(6): p. 2098-2108.
16. Rosenzweig, R., et al., *The Hsp70 chaperone network.* Nat Rev Mol Cell Biol, 2019. **20**(11): p. 665-680.
17. Havalova, H., et al., *Mitochondrial HSP70 Chaperone System-The Influence of Post-Translational Modifications and Involvement in Human Diseases.* Int J Mol Sci, 2021. **22**(15).
18. Patterson, R.L., et al., *Inositol 1,4,5-trisphosphate receptor/GAPDH complex augments Ca2+ release via locally derived NADH.* Proc Natl Acad Sci U S A, 2005. **102**(5): p. 1357-9.
19. Shoshan-Barmatz, V., Y. Krelin, and A. Shteinfer-Kuzmine, *VDAC1 functions in Ca(2+) homeostasis and cell life and death in health and disease.* Cell Calcium, 2018. **69**: p. 81-100.
20. Pazarentzos, E., et al., *IkappaBetaalpha inhibits apoptosis at the outer mitochondrial membrane independently of NF-kappaB retention.* EMBO J, 2014. **33**(23): p. 2814-28.
21. Neganova, I., et al., *An important role for CDK2 in G1 to S checkpoint activation and DNA damage response in human embryonic stem cells.* Stem Cells, 2011. **29**(4): p. 651-9.
22. Luckheeram, R.V., et al., *CD4(+)T cells: differentiation and functions.* Clin Dev Immunol, 2012. **2012**: p. 925135.

23. Rosencrans, W.M., et al., *alpha-Synuclein emerges as a potent regulator of VDAC-facilitated calcium transport.* Cell Calcium, 2021. **95**: p. 102355.
24. Rostovtseva, T.K., et al., *alpha-Synuclein Shows High Affinity Interaction with Voltage-dependent Anion Channel, Suggesting Mechanisms of Mitochondrial Regulation and Toxicity in Parkinson Disease.* J Biol Chem, 2015. **290**(30): p. 18467-77.
25. Shteinfer-Kuzmine, A., et al., *A VDAC1-Derived N-Terminal Peptide Inhibits Mutant SOD1-VDAC1 Interactions and Toxicity in the SOD1 Model of ALS.* Front Cell Neurosci, 2019. **13**: p. 346.
